# Digital biomarkers for interstitial glucose prediction in healthy individuals using wearables and machine learning

**DOI:** 10.1038/s41598-025-14172-z

**Published:** 2025-08-18

**Authors:** Xinyu Huang, Franziska Schmelter, Christian Seitzer, Lars Martensen, Hans Otzen, Artur Piet, Oliver Witt, Torsten Schröder, Ulrich L. Günther, Lisa Marshall, Marcin Grzegorzek, Christian Sina

**Affiliations:** 1 Institute of Medical Informatics, University of Luebeck, Lübeck, Germany; 2 Institute of Nutritional Medicine, University of Luebeck and University Medical Center Schleswig-Holstein, Lübeck, Germany; 3 Perfood GmbH, Research and Development, Lübeck, Germany; 4 Institute of Chemistry and Metabolomics, University of Luebeck, Lübeck, Germany; 5 Institute of Experimental and Clinical Pharmacology and Toxicology, University of Luebeck, and University Medical Center Schleswig-Holstein, Lübeck, Germany; 6 Center of Brain, Behavior and Metabolism (CBBM), Lübeck, Germany; 7 German Research Center for Artificial Intelligence (DFKI), Lübeck, Germany; 8 Fraunhofer Research Institution for Individualized and Cell-Based Medical Engineering (IMTE), Lübeck, Germany

**Keywords:** Non-invasive CGM, Interstitial glucose prediction, Personalized nutrition, Machine learning, Engineered biomarker, Wearables, Public health, Predictive markers

## Abstract

A personalized low-glycemic diet, maintaining stable blood glucose levels, aids in weight reduction and managing (pre-)diabetes and migraines in individuals. However, invasiveness, high cost, and limited lifecycle of continuous glucose monitoring (CGM) devices restrict their widespread use. To address these issues, we investigated machine learning (ML) approaches for glucose monitoring using data from non-invasive wearables. Our study comprised two phases involving healthy participants: The main study included two experimental sessions lasting 7–8 h with two standardized test meals, totaling over 1550 interstitial glucose (IG) measurements with CGM, and high-frequency multimodal data collected by two different non-invasive sensor devices. The follow-up study involved more than 14,400 IG measurements. Using ML approaches, correlations between glycemic measures and sensor data were assessed to estimate the feasibility of accurately predicting personalized IG alterations in real-time. An ensemble feature selection-based light gradient boosting machine (LightGBM) algorithm, omitting the need for food logs, was developed. This algorithm achieved a root mean squared error (RMSE) of 18.49 ± 0.1 mg/dL and a mean absolute percentage error (MAPE) of 15.58 ± 0.09%, demonstrating the feasibility of non-invasive glucose monitoring with high accuracy, which paves the way for novel approaches in the objective prevention of diet-related diseases.

## Introduction

A high postprandial glucose response (PPGR) is a significant contributor to metabolic dysfunction, leading to postprandial hyperinsulinemia, lipogenesis, and visceral obesity^1,2^. Therefore, stable blood glucose (BG) levels are crucial for weight control and preventing risks like metaflammation, insulin resistance, Type 2 diabetes (T2D)^3,4^, and associated conditions such as cardiovascular diseases, renal dysfunction, cancer, and neurodegenerative diseases^5–8^. Further, maintaining glucose homeostasis is essential not only for the prevention of T2D but also for other pathophysiologic conditions such as acne^9^ and migraine^10^, demonstrating a broad range of applications.

Diet influences PPGR, recommending a balanced, plant-based, low-saturated fats, and low-sugar diet^11–13^. However, issuing generalized recommendations to lower PPGRs avoiding glucose spikes is challenging due to the highly individual nature of PPGRs^14,15^. Other PPGR limiting strategies such as ketogenic diets come with other caveats, emphasizing the need for digital postprandial low-glycemic diets (PLGD) for prevention and for individuals dealing with obesity, pre-diabetes, and diabetes^16–18^.

Continuous glucose monitoring (CGM) is commonly used to measure interstitial glucose (IG) levels but has limitations like invasiveness, inconvenience, high cost, and environmental impact. A solution might be the use of non-invasive wearables. First data demonstrate the potential to predict IG values in real-time using physiological parameters and dietary information^19–24^. However, the latter models require the integration of supplementary data of daily dietary intake. This requires strong adherence and commitment from individuals, as self-tracking dietary habits is time-consuming, additionally subjective, and error prone.

Here we asked what kind of approach might limit the disadvantages of both invasive CGM and food diary based PPGR prediction models. Therefore, a two-phase experiment was conducted on metabolic healthy volunteers, with the aim to identify non-invasive sensor modalities for predicting IG values in real-time without relying on time-consuming self-records. The identification of non-invasive sensor modalities in this study represents a significant milestone for monitoring and controlling IG levels and offers the potential for a wider use of personalized approaches in glucose management.

## Results

### Outcomes of correlation analysis

**Fig. 1:**
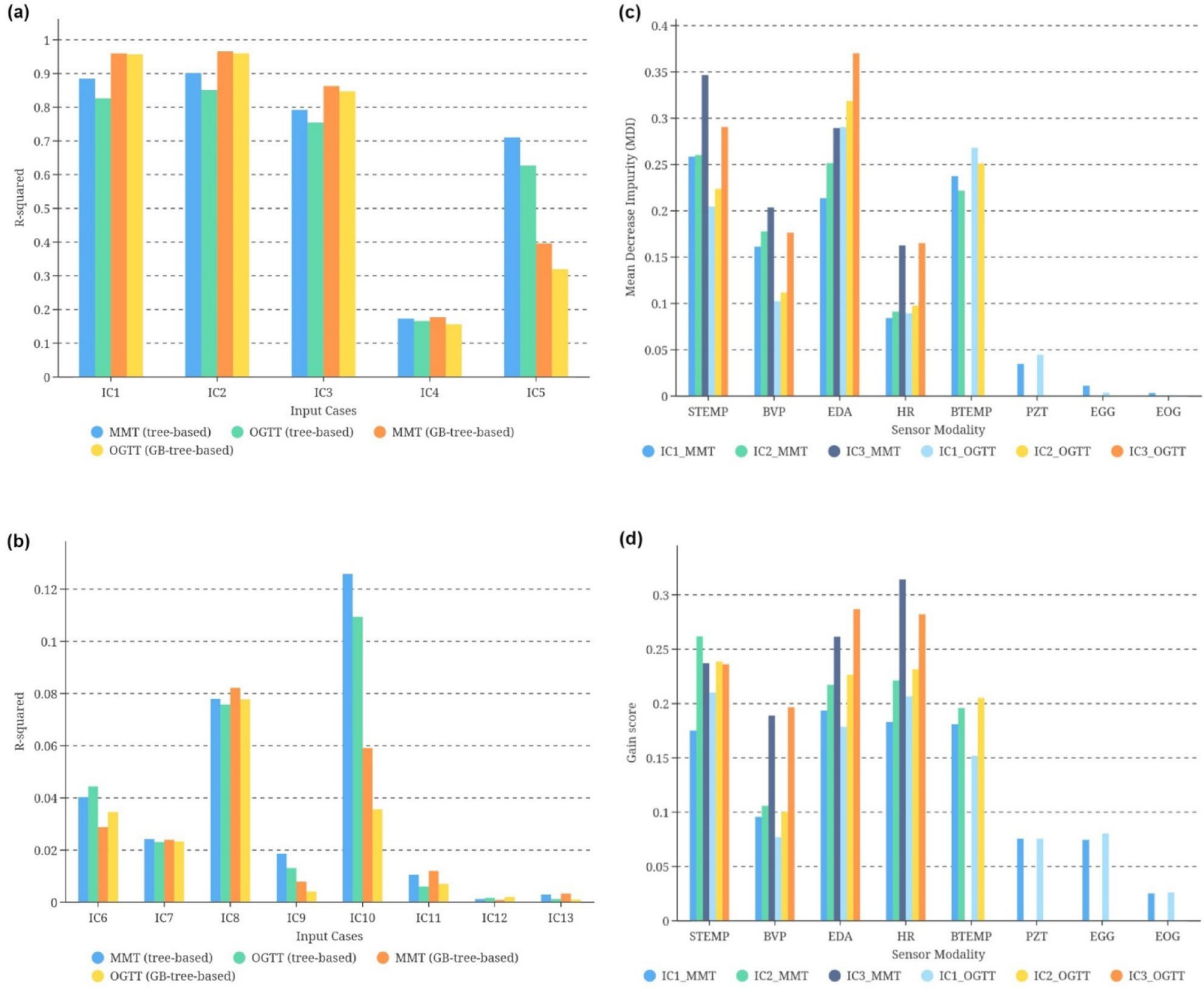
Data-driven correlation ablation test between sensor modalities with IG responses in MMT and OGTT interventions. (**a**) Data-driven correlation ablation test between fused sensor modality with IG responses in MMT and OGTT interventions (two hours for each standardized meal intervention), including IC1—all eight sensor modalities (STEMP, BVP, EDA, HR, BTEMP, PZT, EGG, and EOG); IC2—five sensor modalities (STEMP, BVP, EDA, HR, and BTEMP); IC3—four sensor modalities from Empatica E4 (STEMP, BVP, EDA, and HR); IC4—four sensor modalities from BiosignalsPlux (BTEMP, PZT, EGG, and EOG); IC5—three sensor modalities from Empatica E4 (STEMP, BVP, and EDA). (**b**) Data-driven correlation ablation test between single sensor modality with IG responses in MMT and OGTT interventions, including IC6—single sensor modality STEMP; IC7—single sensor modality BVP; IC8—single sensor modality EDA; IC9—single sensor modality HR; IC10—single sensor modality BTEMP; IC11—single sensor modality PZT; IC12—single sensor modality EGG; IC13—single sensor modality EOG. (**c**) Tree-based sensitivity testing of each sensor modality with IG responses after MMT and OGTT interventions (two hours for each standardized meal intervention) (MDI) based on IC1, IC2, and IC3. (**d**) GB-tree-based sensitivity testing of each sensor modality with IG responses after MMT and OGTT interventions (Gain) based on IC1, IC2, and IC3.

Figure 1 demonstrates the testing of different input cases (IC), including combinations (Fig. 1a) and single sensor modalities (Fig. 1b) (for the definition of the different combinations of IC, see STable 1), using tree-based and GB-tree-based approaches, with 32-fold cross-validation in terms of R-squared (R^2^). To determine the individual sensitivities of each sensor modality, their mean decrease in impurity (MDI)^25^ and Gain scores^26^ were calculated for different ablation test settings, as illustrated in Fig. 1c, d, respectively. For details, based on Fig. 1b, it can be observed that there are no linear nor nonlinear correlations between single sensor modality and responses in IG, with R^2^ values consistently below 0.15. Only combinations of multiple sensor modalities (Fig. 1a) can effectively reflect the approximate changes in IG. In particular, the IC2 input combination of skin temperature ‘STEMP’, blood volume pulse ‘BVP’, heart rate ‘HR’, electrodermal activity ‘EDA’, and body temperature ‘BTEMP’ displayed the highest correlations between sensor data and alterations in IG. Within two hours after consuming mixed meal test (MMT) (Fresubin® 2 kcal), tree-based and GB-tree-based algorithms demonstrated high R^2^ values of 0.90 and 0.96, while oral glucose tolerance test (OGTT) consumption within the same timeframe yields R^2^ values of 0.85 and 0.96. Furthermore, when using all eight sensor modalities, the tree-based algorithm has an average distribution fit of over 0.85 and the GB-tree-based algorithm achieves over 0.95 (Fig. 1a).

However, not every sensor modality contributed equally to reflecting the responses in glucose levels. It is necessary to calculate the importance of each relevant variable. We calculated the relative Gini-MDI and Gain importance of each sensor modality in the IC1, IC2, and IC3 under different meal interventions (Fig. 1c, d). Interestingly, the tree-based analysis revealed that temperature modalities (variables) (STEMP and/or BTEMP) were more sensitive to glycemic responses than other modalities after MMT intake (average MDI: 0.293—STEMP vs. 0.246—EDA). In contrast, EDA monitoring data was more important after OGTT intake (average MDI: 0.327—EDA vs. 0.233—STEMP). In terms of the GB-tree-based analysis, temperature variables, and EDA were generally of similar importance in the monitoring phase after consuming both MMT and OGTT (average Gain in MMT: 0.218—STEMP and 0.223—EDA; average Gain in OGTT: 0.228—STEMP and 0.227—EDA), with more emphasis on the importance of HR (average Gain: 0.24 for MMT and 0.242 for OGTT). Instead, the importance of respiratory rate ‘PZT’, electrooculography ‘EOG’, and electrogastrography ‘EGG’ remained below 0.1 (MDI and Gain) and may interfere with IG prediction, so the degree of fit of IC2 data after the removal of these three sensor modalities was higher than that of IC1. Consequently, the input cases of IC1 and IC2 were further used in the following feature-driven experiments to test the hypothesis in this data-driven correlation ablation test.

### PPGR prediction performances

**Fig. 2:**
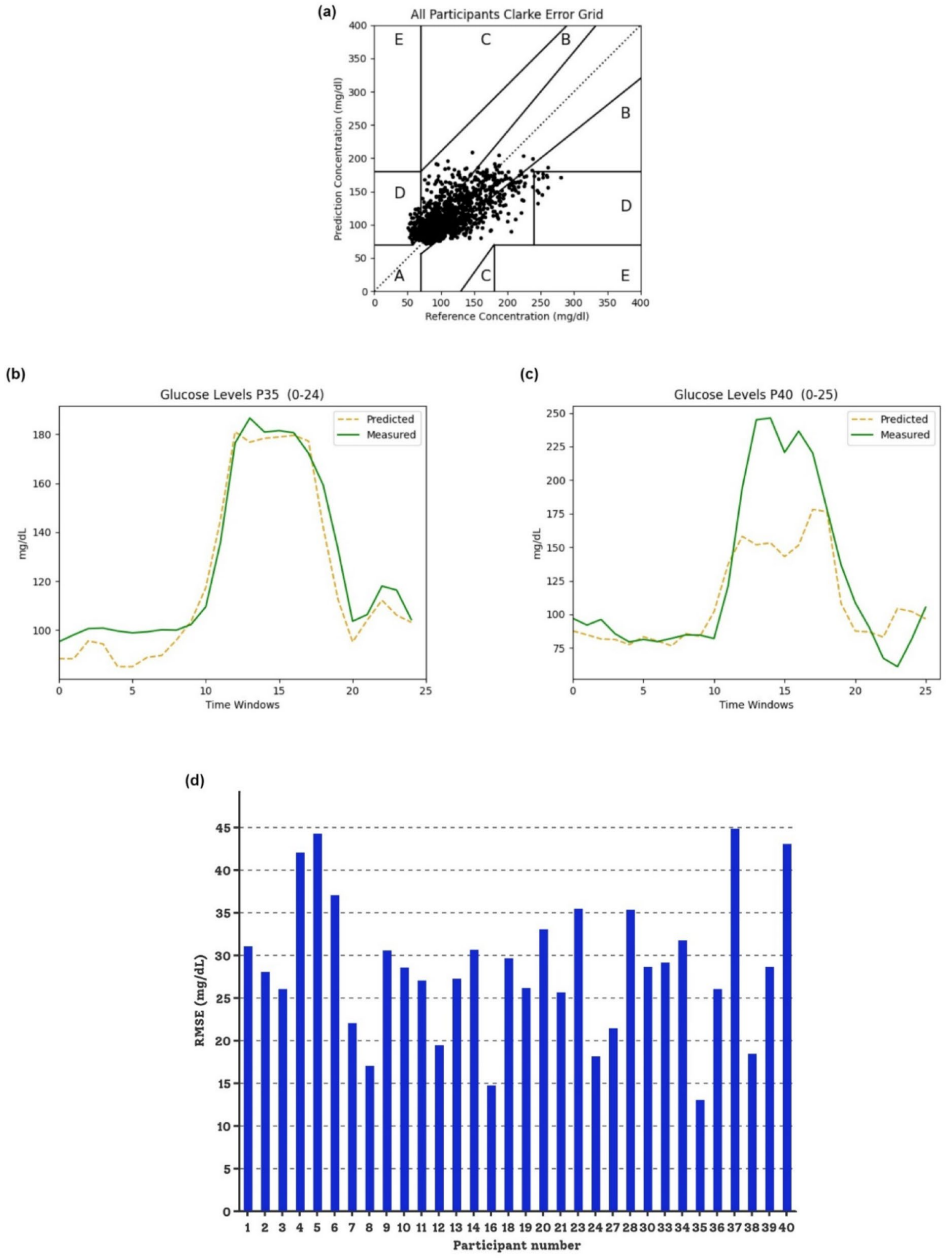
Variability in IG prediction performance using different evaluation metrics across all participants in the main study (IC2). (**a**) Evaluating IG prediction performance for all 32 participants using CEGA. (**b**) Visualized assessment of prediction variance between the IG meter measurements and model-based predicted values for participant #35 (P35) (each point on the X-axis represents a PH of 15 min). (**c**) Visualized assessment of prediction variance between the IG meter measurements and model-based predicted values for participant #40 (P40) (each point on the x-axis represents a PH of 15 min). (**d**) Evaluation of IG prediction performance for each participant based on RMSE (mg/dL).

Based on the findings of the previous correlation ablation test, we performed glucose prediction based on feature engineering (FE)^27^ in terms of the feature-driven way utilizing random forest (RF)^28^, light gradient boost machine (LightGBM)^29^, and long short-term memory (LSTM)^30^, across 32 participants. A total of about 56 measured IG meter values of each participant (seven hours of data per day for two days, including at least 2-h washout phase) were matched with preprocessed 20 Hz continuous time-series data (under test conditions IC1 and IC2) joined with user demographics for the prediction trial. The proposed models were trained and validated with the leave-one-participant-out cross-validation (LOPOCV)^31^ to eliminate the factor of personal deviation. In addition to sensor data, personal demographic data, phase marker (MMT, OGTT, and washout), and two time-domain features (as provided in STable 2) were further used to develop the glucose prediction population models. Referring to Table 1A, FE-based LightGBM and RF models outperformed deep-learning-based LSTM. Although the performance difference between RF and LightGBM models is not significant, the RF model with an ensembled feature selection (FS) strategy that integrates the advantages of the Recursive Feature Elimination (RFE) and Boruta (called BoRFE), configured with a prediction horizon (PH) of 15 min under IC2 (number of sessions (SN) = 5, i.e., STEMP, BVP, EDA, HR, and BTEMP), validated with LOPOCV, had an average root mean squared error (RMSE) of 26.83 ± 0.03 mg/dL and mean absolute percentage error (MAPE)^32^ of 18.76 ± 0.04%. Additionally, we visualized the performance using Clarke error grid analysis (CEGA)^33^ (Fig. 2a). Most points fall into regions A and B, indicating that the deviation of the predicted glucose values compared to the CGM reference is less than 20% or would not result in inappropriate clinical management. A few prediction errors appeared in the right and left D regions (< 3.58%), which belong to the hyper- and hypoglycemia data points. Overall, in our young and healthy cohort only 7.91% of the glucose values belong to level 1 hypoglycemia (< 70 mg/dL) and 0.61% belong to level 2 (< 54 mg/dL) with the lowest glucose level of 52 mg/dL. Although hypoglycemia occurs, these are short-term events that can be compensated for by the body’s own regulation, and there are no clinically relevant cases of hypoglycemia over a longer time period. Therefore, there are no cases related to level 3 where medical help was necessary (see Table 2, Phase I).

**Table 1:** Interstitial glucose prediction performance comparison: In the main study (A.), feature-driven performance comparison between RF, LightGBM, and LSTM models with different feature selection strategies based on IC1 (eight sensor modalities / SN = 8) and IC2 (five sensor modalities / SN = 5) test conditions evaluated by LOPOCV using mean RMSE (mg/dL) with prediction deviations <  ± 0.07 mg/dL and MAPE (in %) with prediction deviations <  ± 0.06%; In the follow-up study (B.), comparison of glucose prediction performance using BoRFE strategy with LOPOCV among three different models (RF, LightGBM, and LCE) targeted at all five participants (FUIC1) , four participants (excluding P2) (FUIC2), and five participants with removing outliers (< 70 mg/dL) (FUIC3) based on IC2: five sensor modalities (STEMP, BVP, EDA, HR, and BTEMP) evaluated by mean RMSE (mg/dL) with prediction deviations <  ± 0.11% and mean MAPE (in %) with prediction deviations <  ± 0.18%. The highest performances in each study phase are highlighted in bold and underlined.

	Models	PH = 5 min				PH = 15 min			
		SN = 8 (IC1)		SN = 5 (IC2)		SN = 8 (IC1)		SN = 5 (IC2)	
		RMSE	MAPE	RMSE	MAPE	RMSE	MAPE	RMSE	MAPE
(A). Main study	LightGBM + Boruta	28.40	19.62	28.25	19.65	27.72	19.91	27.30	18.83
	LightGBM + RFE	28.47	19.77	28.40	19.91	27.93	19.92	33.66	24.10
	LightGBM + BoRFE	34.54	24.28	35.73	24.78	27.55	19.43	27.29	18.91
	LightGBM without FS	28.51	19.76	28.45	20.04	29.83	21.40	29.09	20.74
	RF + Boruta	28.55	20.04	28.57	20.18	27.35	19.27	27.07	18.92
	RF + RFE	27.96	19.97	35.20	24.51	34.00	23.62	34.43	24.30
	RF + BoRFE	33.78	24.02	34.59	24.34	27.69	19.78	**26.83**	**18.76**
	RF without FS	28.61	20.02	29.03	20.43	31.11	22.39	29.90	21.51
	LSTM	33.26	22.69	30.39	21.08	61.58	49.65	65.04	51.94

				FUIC1 (5 participants)		FUIC2 (4 participants [excluding P2b])		FUIC3 (5 participants [remove outliers < 70 mg/dL])	
				RMSE	MAPE	RMSE	MAPE	RMSE	MAPE
(B). Follow-up	RF + BoRFE			18.87	16.36	16.95	12.96	**18.81**	16.38
	LightGBM + BoRFE			**18.49**	**15.58**	**16.89**	**12.79**	18.96	**15.29**
	LCE with FE			19.93	16.55	16.97	12.99	19.89	16.67

**Table 2:** Proportion for IG values in the dataset.

Phase		Lowest IG value (mg/dL)	Level 1 [< 70 mg/dL] (in %)	Level 2 [< 54 mg/dL] (in %)
I. pilot study	P2		6.06	0
	P3		15.94	0
	P6		4.35	0
	P7		5.80	0
	P10		10.14	0
	Others	52	8.04	0.48
	Total (in %)		7.91	0.61
II. follow-up study	P2	52	15.56	1.03
	P3		5.73	0.44
	P6		0.98	0
	P7		0.06	0
	P10		0.80	0
	Total (in %)		4.90	0.31

Meanwhile, Fig. 2b–d illustrate the prediction bias between individuals based on the RMSE (i.e., prediction instances of two specific participants and an overall comparison of all 32 individuals). Interestingly, already in a small dataset without detailed information on food/meal components and activities, our proposed FE-based population model shows promising results comparable to the performance of other modern studies^22,34^, although some interindividual differences were noted.

### Validation test results

**Fig. 3:**
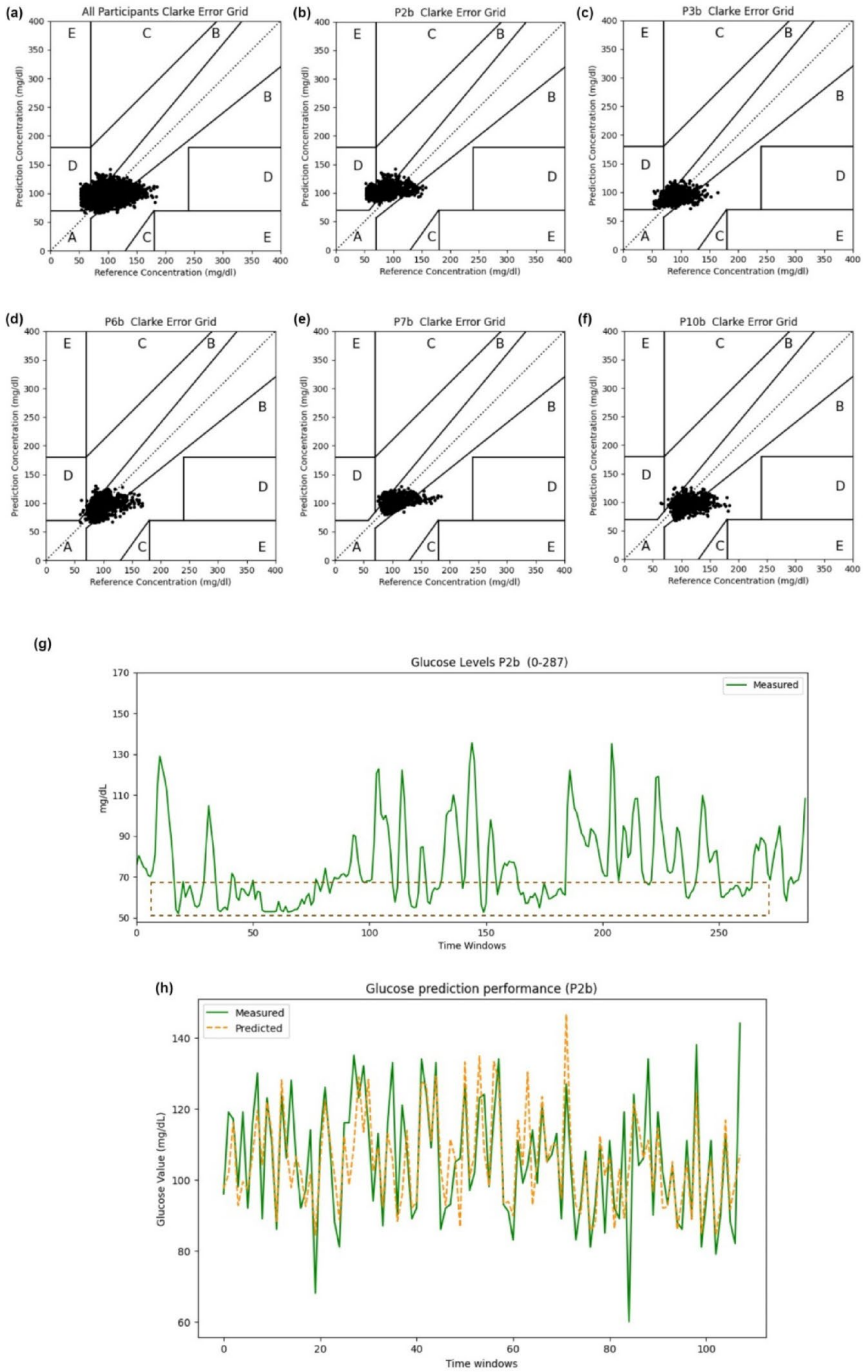
IG prediction performances evaluated by CEGA and intuitive visualized predictive curves across participants in the follow-up (FUIC1, five participants) study (PH = 15 min). (**a**) Glucose prediction performance based on data from all five participants assessed by CEGA with PH = 15 min using the BoRFE-based LightGBM. (**b**) Glucose prediction performance based on data from participant #2 (P2b) assessed by CEGA with PH = 15 min using BoRFE-based LightGBM. (**c**) Glucose prediction performance based on data from participant #3 (P3b) assessed by CEGA with PH = 15 min using BoRFE-based LightGBM. (**d**) Glucose prediction performance based on data from participant #6 (P6b) assessed by CEGA with PH = 15 min using BoRFE-based LightGBM. (**e**) Glucose prediction performance based on data from participant #7 (P7b) assessed by CEGA with PH = 15 min using BoRFE-based LightGBM. (**f**) Glucose prediction performance based on data from participant #10 (P10b) assessed by CEGA with PH = 15 min using BoRFE-based LightGBM. (**g**) Glucose meter measurements (ground truth) were obtained from Abbott Freestyle Libre 2 to show the first three days of P2b’s data with highlighted hypoglycemic conditions (by a red dashed frame, value < 70 mg/dL) (each point on the x-axis corresponds to a 15-min prediction horizon). (**h**) Visualization of the interstitial glucose levels prediction real matching curve between the ground truth (CGM values) and the predicted values for 24 h in P2b.

We applied the previously proposed glucose prediction models (RF and LightGBM) in our follow-up study (five participants, over ten days, daily life data including five sensor modalities and IG responses without involving their food diaries) to validate the feasibility of the population model in real-world scenarios. In particular, we further decided to test an additional model called Local Cascade Ensemble (LCE)^35^, which combines the advantages of both tree-based and GB-tree-based techniques. Table 1B demonstrates that LightGBM achieved superior performance compared to the other two models for all five participants in the follow-up study input case 1 (FUIC1), utilizing the BoRFE FS strategy and LOPOCV. The performance of the system was exceptional. Firstly, it achieved an average RMSE of 18.49 ± 0.1 mg/dL, an average MAPE of 15.58 ± 0.09%, and less than 2.15% of prediction deviations in the left D regions (Fig. 3a). Secondly, it achieved an average RMSE of 16.89 ± 0.03 mg/dL and an average MAPE of 12.79 ± 0.08% for four participants (FUIC2, excluding P2b to eliminate the risk of undiagnosed prediabetes symptoms, such as spontaneous hypoglycemia shown in the dashed box in Fig. 3g). Thirdly, it achieved an average RMSE of 18.96 ± 0.09 mg/dL and an average MAPE of 15.29 ± 0.08% for all five participants by removing outliers (< 70 mg/dL, FUIC3). In total, 4.9% of the glucose values of this dataset belong to level 1 hypoglycemia, 0.31% belong to level 2, without cases related to level 3 (see Table 2, Phase II). For its training speed advantage and highlighting the function of HR features, which may be utilized for hypoglycemia prediction, while time-domain features, EDA and TEMP remained consistently superior. The overall and subject-specific performance visualizations of IG prediction using the CEGA under FUIC1 and FUIC2 conditions are depicted in Fig. 3a–h and in SFig. 1a–e, respectively. These optimized prediction errors demonstrated the effectiveness of the domain-specific FE and ensemble FS strategy, further confirming the feasibility of predicting the IG fluctuations of healthy individuals in their daily lives using only sensor data, without the need for food, metabolic, and activity data.

### Feature significance

**Fig. 4:**
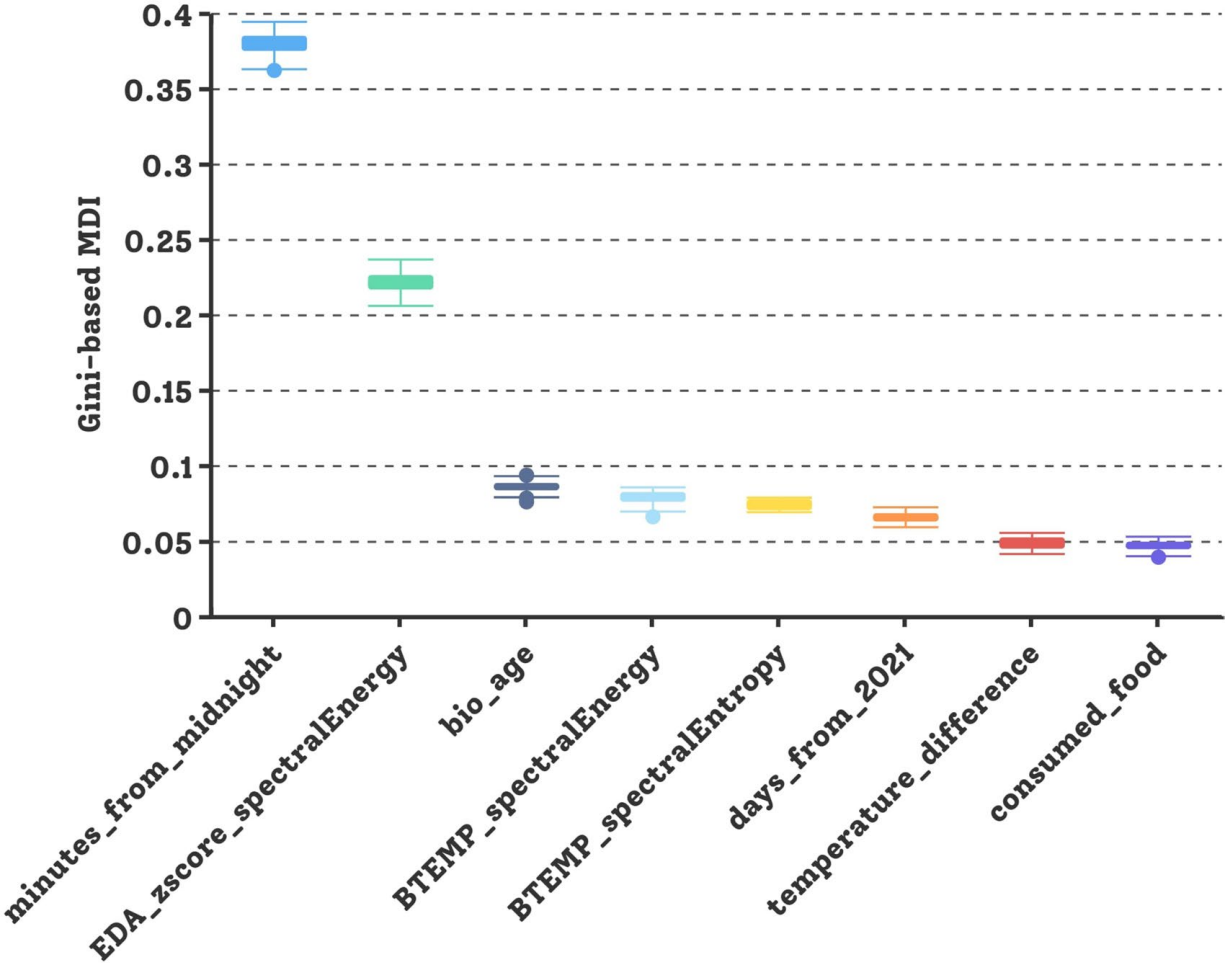
Gini-based FR in glucose prediction with BoRFE based on IC2 test conditions (SN = 5 and PH = 15 min) in main study.

To streamline IG prediction and enhance interpretability, FS and feature ranking (FR) methods were employed. The main study assessed each feature’s contribution using Gini-based MDI importance in the RF regression model, revealing seven significant features (MDI > 0.03) in the context of IC2, including time-domain features, EDA, BTEMP, phase marker, and demographics. The variance of the feature importance among each participant in LOPOCV is depicted in Fig. 4.

**Fig. 5:**
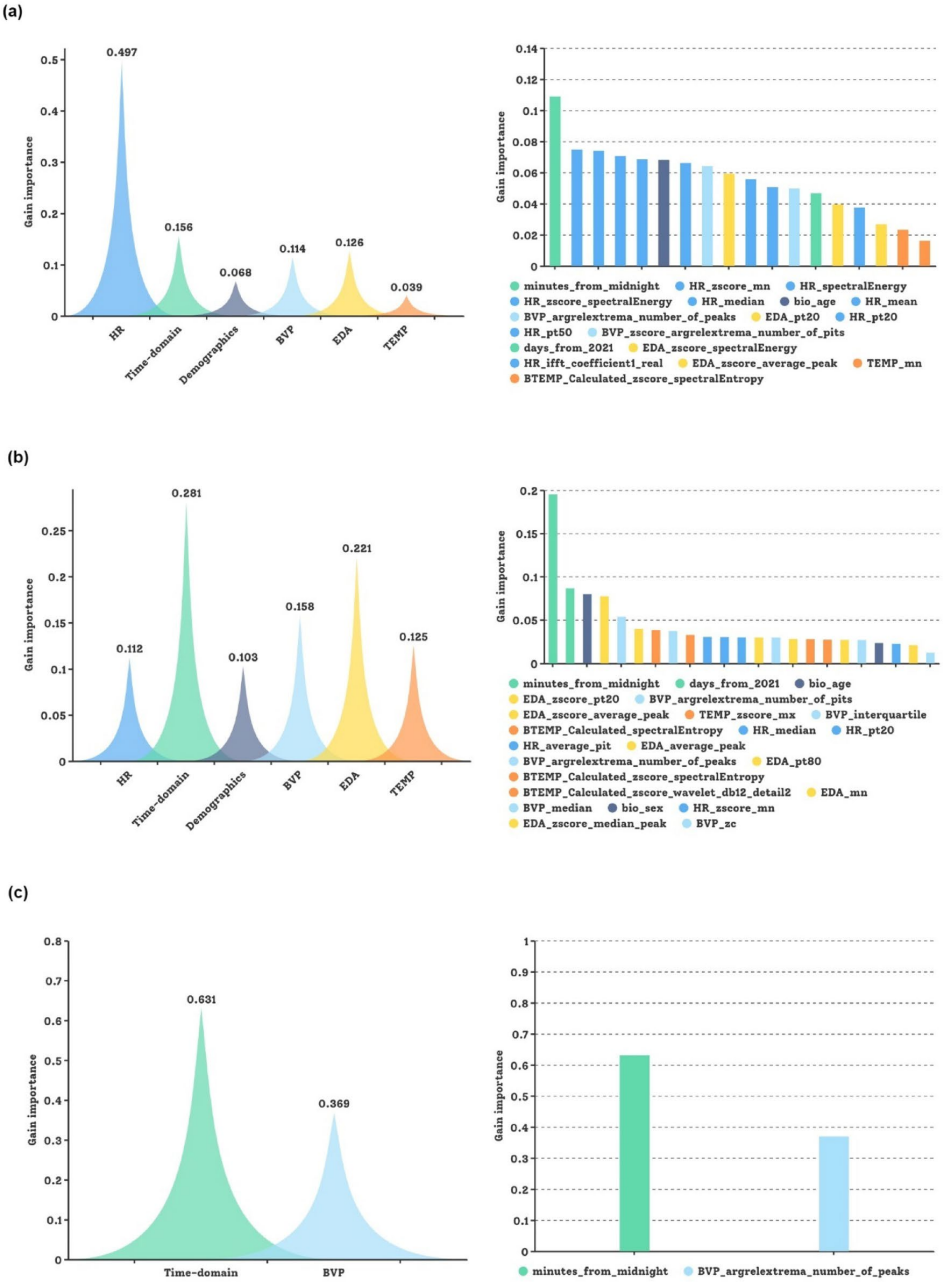
Feature importance rankings on the feature main/subcategory level in the follow-up study. (**a**) Intuitive bar plots depicting feature importance rankings on the main feature category and feature subcategory level for FUIC1 (IG prediction using BoRFE- and LOPOCV-based LightGBM configurated with PH = 15 min). (**b**) Intuitive bar plots depicting feature importance rankings on the feature main category and feature subcategory level for FUIC2 (IG prediction using BoRFE- and LOPOCV-based LightGBM configurated with PH = 15 min). (**c**) Intuitive bar plots depicting feature importance rankings on the feature main category and feature subcategory level for FUIC3 (IG prediction using BoRFE- and LOPOCV-based LightGBM configurated with PH = 15 min).

For our follow-up study, the same feature categories except phase markers were utilized for glucose prediction with LightGBM based on FUIC1, FUIC2, and FUIC3. In FUCI1, after conducting the ensemble FS and FR, 18 features were ranked in descending order by GAIN importance. As illustrated in Fig. 5a, the chosen features in the study, such as the measures of time-domain ‘minutes_from_midnight’ and ‘days_from_2021’; demographics ‘bio_age’; sensor data ‘EDA_zscore_sprectralEnergy’ and ‘BTEMP_sprectralEntropy’, also occupy the same relatively important position in the FR of the follow-up study. Here the importance of HR is significantly emphasized with an average of 0.497, followed by time-domain features (0.156), EDA (0.126), BVP (0.114), demographics (0.068), and TEMP (0.039). In contrast, in FUIC2, the importance of HR-related features decreased to 0.112, whereas the importance of EDA- and TEMP-related features increased to 0.221 and 0.125, respectively, which is consistent with our outcomes from the main study. Moreover, the significance of the time-domain features in the follow-up study is rationally diluted (0.281) based on the substantial increase in the dimension of the experimental data. When analyzed in conjunction with the specificity of the hypoglycemia data in P2b, it can be observed that HR has a significant function in predicting outlier values (compare these features importance in Fig. 5a, b). Nevertheless, in Fig. 5c, after FS, only two categories of features ‘minutes_from_midnight’ and ‘BVP_argrelextrema_number_of_peaks’, were retained to be responsible for the glucose prediction output. Apparently, the strategy of simply removing outliers is not recommended, as the nonlinear correlations between sensor data and glucose responses are thereby eliminated.

### Generality test

To verify the generality of the proposed model, a comparative validation experiment was performed on a public dataset^22,36,37^, i.e., Big-ideas-glycemic-wearable (BiGW), containing 16 participants aged 40–69 who wore the Empatica E4 over eight days. All participants had high fasting blood glucose levels or prediabetes, which resulted in mildly elevated blood glucose levels and an increased risk of T2D. As shown in Table 3, our model acted consistently under all four different input modal conditions and even achieved the best performance (with an RMSE of 13.59 mg/dL) for the IG prediction in the CEGA evaluation considering regions A (90.60%), B (9.11%), and D (0.29%) without leveraging the diet and exercise (tri-axial accelerometry) logs. It further validates the superiority and robustness of the proposed LightGBM model and metaphorically tests its predictive compatibility for glucose outliers.

**Table 3:** Model’s generality test on the public BiGW dataset under four sensor input conditions using LightGBM with PH = 15 min and resampling rate of 20 Hz evaluated by RMSE (mg/dL), MAPE, and CEGA (% in each prediction areas) based on the LOPOCV (The highest performances are highlighted in bold and underlined).

Sensor modalities (BoRFE-based LightGBM)	RMSE (mg/dL)	MAPE	CEGA (%)					
			Area A	Area B	(A + B)	Area C	Area D	Area E
Without food logs	14.70	8.53	**91.02**	8.62	99.64	0	0.36	0
Without activity logs	15.64	10.11	90.04	9.42	99.46	0	0.54	0
Without food and activity logs	**13.59**	7.99	90.60	9.11	**99.71**	0	**0.29**	0
All sensor modalities	14.01	**7.78**	89.45	**9.73**	99.18	0	0.82	0

## Discussion

Our study aimed to predict glucose levels based on non-invasive sensor data and investigated their correlation with standardized meal intakes by identifying physiological factors. To obtain standardization, the glucose responses to two intervention conditions, MMT and OGTT, were investigated. Our results revealed skin temperature (STEMP), body temperature (BTEMP), blood volume pulse (BVP), electrodermal activity (EDA), and heart rate (HR) as the strongest correlations with IG alterations. Their relationships were complex and nonlinear, indicating that a single modality could not capture glucose responses accurately.

Based on these initial findings, we developed an ML algorithm that uses hand-crafted features to predict PPGR based on seven hours of sensor data per intervention day. Unlike some studies^22,34^ that exclude glucose measurements above the upper-risk threshold, i.e., > 180 mg/dL, we retained these measurements to accurately represent the effect of high-glycemic diets on IG levels. RF algorithm with an ensemble BoRFE feature selection strategy demonstrated reliable performance with a small average measurement error. Convincingly, features associated with EDA and BTEMP played crucial roles in the performance of IG prediction. Additionally, the time-domain feature for eating timestamps ‘minutes_from_midnight’ and the seasonal feature ‘day_from_2021’ contributed significantly to calibrating prediction errors. Seasonal ambient temperature previously was previously shown to impact participants’ body, core, and skin temperatures^38^.

The observed relationships as well as the physiological and mechanistical framework between sensor-derived attributes and postprandial glucose fluctuations are complex. A study by Jayarathna et al.^39^ investigating the detection of nocturnal hypoglycemia using EOG revealed significant differences in saccade velocity patterns between hypoglycemic and normoglycemic conditions. Hypoglycemia can impair eye movement function, by reducing saccade velocity, by inhibiting the energy metabolism of the retina. According to research^40^, there is a complex connection between glucose levels and PZT. Insulin infusion increases respiratory rate in healthy young men, even when glucose levels are kept at euglycemic values. Oral glucose consumption boosts EGG dominant frequency and power ratio while slowing gastric emptying^41^. Although previous studies have shown that EOG, EGG, and PZT are physiologically correlated with IG fluctuations, the actual impact of these indicators in our pilot study was limited due to experimental constraints.

Elevations in skin and core body temperature could be a result of postprandial thermogenesis, a metabolic process that is accelerated by sympathetic activation mediated by insulin^42^. Temperature patterns may be further modulated by peripheral vasodilation during glucose absorption, and these effects may be intensified by ambient thermal stress (derived by ‘day_from_2021’) through altered cutaneous blood flow^43^. Sympathetic tone, which is correlated with EDA, directly affects the production of glucose in the liver through β-adrenergic pathways^44,45^. Heart rate variability and blood volume pulse are sensitive to baroreflex adaptations to fluid redistribution during glycemic excursions and insulin-induced vasodilation. These relationships’ nonlinearity is consistent with the autonomic nervous system’s known biphasic reaction to abrupt changes in blood sugar levels^46^. Furthermore, consistent with the physiology of the dawn phenomenon, the predictive power of circadian timing features (‘minutes_from_midnight’) certainly originates from diurnal variations in insulin sensitivity, such as glucocorticoid and growth hormone surges during early morning hours that counteract insulin action^47^.

To sum up the results of our main study, our ML algorithm showed minimal errors during meal interventions across 32 participants, promising the potential for wearable technology in glucose monitoring. However, glucose prediction errors varied among individuals, dependent upon (a) individual responses of the healthy non-diabetic cohort with low hypoglycemia rates, (b) other biological issues such as young age, and (c) technical issues such as data quality.

To evaluate the practicality of our population approaches in real-world prediction scenarios, we extended the experiment duration to ten days in the follow-up study (FU). During this period, we blinded the meal interventions and collected only wearable sensor data and IG responses from the participants. Our advanced ML algorithm, predicting PPGR based on non-invasive sensor data without using food logs, performed remarkably well, achieving lower average errors than previous studies. However, it’s important to note that our algorithm focused on predicting PPGR in healthy individuals and may not accurately predict CGM^48^ values outside the normal range due to limited data points.

We observed that one participant (P2b) had many CGM values outside the normal range (dropped below 70 mg/dL or exceed 180 mg/dL), impacting the algorithm’s performance. These outliers cannot be easily excluded since we observed a certain tendency and correlation between adjacent IG values. Simply removing these outliers can disrupt the overall coherence and logic of the data, leading to significant mispredictions, particularly when utilizing LOPOCV. Therefore, we tend to exclude this participant, allowing the performance of the algorithm to be improved to bring it in line with the performance of widely used CGM devices. According to the manufacturer’s specification the Abbott Freestyle Libre sensor has a mean absolute relative difference (MARD) of 9.3%^49^, which is set at a threshold of 100 mg/dL. Above this threshold, the error for a valid continuous glucose measurement can expand up to ± 20%, while if the meter value is below this threshold, the error is set at ± 20 mg/dL. Our novel algorithm’s robustness under a well-designed research architecture, with an average mean RMSE of 18.49 ± 0.1 mg/dL or 16.89 ± 0.03 mg/dL for the naive case (FUIC2) was comparable to previous studies^22,34,50–57^ (see Table 4). For instance, Geng et al.^50^ employed a cross-correlation function to screen the features for the glucose prediction. While the study achieved remarkable results, it also has certain limitations. The stringent dietary control, the discomfort associated with finger-stick glucose monitors, and the constraints of basic signal similarity calculation algorithms when dealing with extensive data have presented challenges for their practical implementation in real-world scenarios. Similarly, a study by van den Brink^34^ used XGboost to predict glucose levels and achieved impressive performance with an MAE of 11.17 mg/dL (0.62 mmol/L). However, their study subjects were healthy individuals, where prediabetes were not specifically excluded. Among the 24 participants, 16 had fasting blood glucose levels below 5.6 mmol/L, and they excluded almost all extreme outliers from their analysis. Furthermore, the assessment criteria in both studies^34,50^ were internally established for the same participant in both the training and test set. This could potentially result in performance discrepancies among participants. Furthermore, a study by Bent et al.^22^ used a gradient-boosted population model with LOPOCV resulting in a RMSE of 21.22 ± 4.14 mg/dL and MAPE of 14.33 ± 3.25% in 16 individuals with high normal blood glucose or prediabetes. The experimental framework, which uses a 24-h rolling baseline to predict glucose deviations, may only be applicable to populations with irregular lifestyles to a limited extent. Furthermore, the exclusive use of quantitative assessments without supplementary qualitative validation raises uncertainties regarding the actual effectiveness and clinical applicability of the model. Studies like those conducted by Bent^22^ and van den Brink^34^ also integrated additional data such as food diary, activity records, and sleep assessment to support their glucose prediction models. In contrast, the novel findings of our work included data-driven correlation examinations between sensor data acquired with non-invasive wearables and IG metabolism in two types of meal interventions and obtained accurate feature-driven glucose prediction performances based on an initial constrained dataset (main study). In particular, we proposed a gradient-based approach associated with novel ensemble FE methods that can achieve robust glucose prediction performances in real-time daily life scenarios for healthy individuals without the support of the food log, activity, and other metabolic information (follow-up study). Last but not least, the remarkable experimental performances based on the public BiGW dataset demonstrate the generality of our proposed IG prediction model. These outcomes prove the potential for glucose monitoring and management in healthy populations, which can significantly alleviate the need to manually record daily nutritional intake or exercise events.

**Table 4:** Performance comparison with state-of-the-art studies rely on different ML models and experimental cohorts.

Study	Cohorts	Sensor	Glucose reference	Sensor	Model	Testing strategy	Food control	Area	Evaluation metrics				
	(scale)			modality									
									RMSE (mg/dL)	MAPE (%)	MAE (mg/dL)	MARD (%)	CEGA [A + B zones] (%)
Geng et al.^50^	Healthy (6)	CuPro	Roche glucometer	Isy, Ops, TEMP, Hdi	HCF-based SM		Standard tortilla	W	14.61	11.28			100
	+ Diabetes (3)						(90 g)						
Reddy et al.^52^	No human involvement (theoretical simulation)	MicSe		RMS	CCM			E					
Krishnan et al.^55^	Healthy (10)	ArUnoSP	Chemistry analyser	PPG	HCF-based RF			W				1.2–9.4	
Bent et al.^22^	Pre-diabetes (16)	E4	Dexcom G6 (CGM)	EDA, TEMP, HR, Acc	HCF-based XGBoost	LOPOCV	Efy, Tf, FI	W	21.22	14.33			
Shokrekhodaei et al.^57^	in-vitro simulation	Triad AS7265x, SparkF	D-Glucose simulation	Visible and Near-Infrared (NIR) Spectroscopy	FFNN	fivefold CV			11.1				98.75
von den Brink et al.^34^	Healthy (24)	Philips Elan	Abbott FreeStyle Libre Pro (CGM)	PPG, Acc	HCF-based XGBoost	tenfold CV	FI	W	13.5–16.2		11.17		
							(HowAmI)						
Agrawal et al.^53^	Mixed cohort (99)	iGLU	Fingerstick	NIR, PPG, RaS, OCT	HCF-based DT			F	9.14			10.64	100
Aloraynan et al.^54^	in-vitro simulation	PAS system	D-Glucose simulation	Bio-IScopy, EleSen, MeH	HCF-based RSS								100
Satter et al.^56^	Mixed cohort (5)	TMD 3719	CareSens II Plus	PPG	HCF-based CBT		Fasting (8 -10 h before measurements)	W					> 90
Huang et al.^51^	Healthy (5)	E4	Abbott Freestyle Libre 2 (CGM)	EDA, TEMP, BVP, HR	BiLSTM	LOPOCV		W	13.42	12			96.99
Our study	Healthy (32 + 5), pre-diabetes (16)	E4	Abbott Freestyle Libre 2 (CGM)	EDA, TEMP, BVP, HR	BoRFE-based LightGBM	LOPOCV	Pilot: OGTT, MMT;	W	18.49	15.58			99.71
							Follow-up: NA						

All study cases are listed in ascending order by year. Handcrafted feature = HCF; Decision tree = DT; Near-Infrared Spectroscopy = NIR; tri-axial accelerometry = Acc; Bidirectional LSTM = BiLSTM; Feedforward Neural Network = FFNN; Self-model = SM; Cole–cole model = CCM; Reflected microwave signals = RMS; Wrist = W; Earlobe area = E; Finger = F; Humidity = Hdi; Optical properties = Ops; Impedance spectroscopy = Isy; Customized prototype = CuPro; Microwave-based Sensor = MicSe; Arduino UNO microcontroller and silicon photodiode = ArUnoSP; Empatica E4 = E4; Mid-infrared photoacoustic spectroscopy = PAS; Triad AS7265x Spectroscopy Sensor = Triad AS7265x; SparkFun RedBoard Qwiic = SparkF; Raman Spectroscopy = RaS; Optical Coherence Tomography = OCT; Random Subspace Sampling = RSS; CatBoost = CBT; Eating frequency = EFy; Timing = Tf; Types of food Intervention = FI; Bio-impedance spectroscopy = Bio-IScopy; Electromagnetic sensing = EleSen; Metabolic heat conformation = MeH.

Our study highlights the potential of non-invasive glucose monitoring in healthy populations, reducing the need for manual recording of daily nutrition or exercise. However, our current approach is limited to healthy participants and a short testing period of 10 days, although the peak surveillance sensitivity to outliers was tested on a small public dataset, it may remain flawed^58^. Furthermore, exploring mechanistic links between non-invasive sensor data and causal pathways is challenging. Addressing this, future research should broaden datasets in real-life, validating algorithms with diverse health conditions like obesity, metabolic disorders, prediabetes, and diabetes. Including various IG values and corresponding sensor data will capture outliers with the aim to apply the approach in clinical practice^59^ after systematic validation steps. Consideration of complex meals and additional functions for HR sensor modalities, especially in hyperglycemia and hypoglycemia, should be explored. Future research may utilize explainable ML techniques on nutritionally stratified data to better understand the physiological cause of correlations between glucose alterations and sensor modalities without applying invasive measurements. As an instance, comparing feature importance patterns such as EDA and BTEMP across high-carbohydrate versus high-fat meals employing SHapley Additive exPlanations (SHAP) values may reveal diet-dependent autonomic responses, whereas clustering participants’ glucose chronotypes may reveal circadian-aligned temperature and EDA signs. Transfer learning can be applied for public datasets with nutritional metadata, which could help distinguish between meal composition impacts (e.g., sodium-induced BVP changes) and intrinsic glucose regulation mechanisms as well, boosting clinical interpretability while remaining methodologically consistent with our non-invasive strategy.

To summarize, our study contributes to engineering-based glucose prediction in two key directions. Firstly, we identified potential correlations between specific sensor patterns and IG responses. Secondly, we developed a machine-learning algorithm that predicts PPGR based on non-invasive sensor data. Remarkably, our algorithm achieved performance levels comparable to other studies, despite not relying on factors such as food diaries, exercise data, and sleep assessments. This advancement holds significant promise in terms of user-friendliness and practicality for everyday use, as it eliminates the need for complex, subjective, time-consuming, and error-prone documentation.

## Methods

### Study design

Ethical approval for the study was obtained in Germany from the corresponding ethics committee of the University of Luebeck (AZ 21-314 and AZ 2022-550). All participants provided written informed consent in accordance with Good Clinical Practice.

**Fig. 6:**
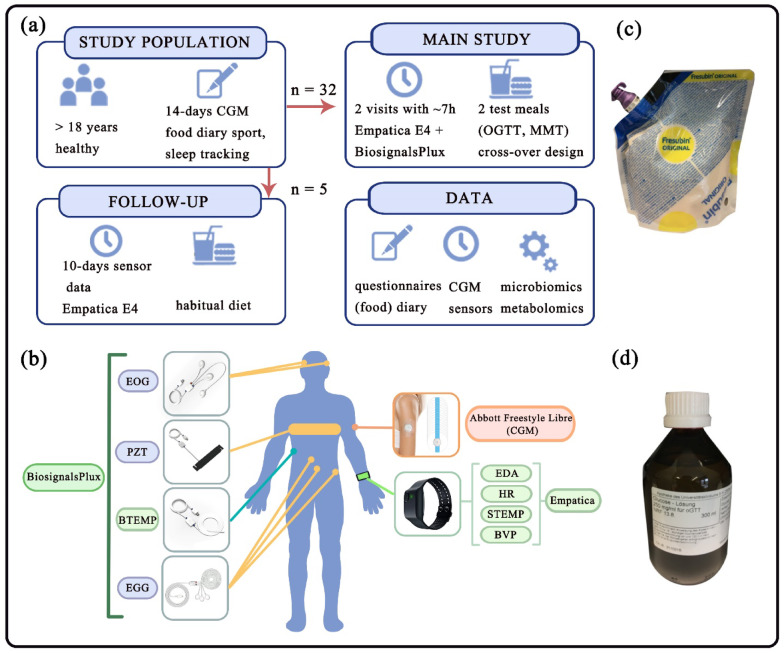
Project design, consisting of a two-phase study (main and follow-up studies) supported by two wearables. (**a**) General study design: a total of 32 healthy adults (women + men) participated in the main study. Their interstitial glucose levels were measured continuously over 14 days, while a food diary was kept via self-report. In the main study, two visits took place at which the Empatica E4 and BiosignalsPlux sensors were worn, and two standardized test meals (mixed meal test—MMT and oral glucose tolerance test—OGTT) were consumed in a cross-over design. In the follow-up study the interstitial glucose levels were recorded continuously for a second time over 14 days in five participants of the study population and the wearable Empatica E4 was worn for ten days under everyday conditions. Taking together the dataset contains information on nutrition, sensor data from Abbott Freestyle Libre continuous glucose monitoring (CGM) and wearables, as well as samples for metabolomics analysis. (**b**) Experimental sensor placements based on three wearables (BiosignalsPlux, Empatica E4, and Abbott Freestyle Libre CGM), including different sensor modalities, i.e., Body temperature ‘BTEMP’, respiratory rate ‘PZT’, electrooculography ‘EOG’, electrogastrography ‘EGG’, skin temperature ‘STEMP’, electrodermal activity ‘EDA’, blood volume pulse ‘BVP’, and heart rate ‘HR’ (efficient sensor modalities for interstitial glucose prediction are highlighted in green and CGM is highlighted in orange). (**c**) Standardized meal intervention—Fresubin® MMT. (**d**) Standardized meal intervention—OGTT.

The clinical study consisted of two phases: (1) a main study and (2) a follow-up study (as illustrated in Fig. 6a). (1) For the main study, 32 healthy women and men aged 20–40 were recruited at the University of Luebeck. Notably, the sample size extended the number of participants of prior studies with comparable methodologies^22,34^. However, formal sample size estimation was not conducted prior to data collection. Due to the lack of available data sets predicting interstitial glucose levels based on non-invasive wearable data without incorporating secondary parameters such as diet or physical activity, the sample size was determined based on practical considerations. In addition to a comprehensive blood panel and the assessment of various liver, kidney, and thyroid parameters, the levels of hemoglobin A1c (HbA1c) were measured to characterize the healthy cohort and specifically confirm that no (pre-)diabetics participated in the study. The HbA1c level was within the normal range at 5.2 ± 0.2%. Throughout the two-week study phase, subjects wore the Abbott Freestyle Libre 2 sensor, on their upper arm, that measured IG levels (mg/dL) continuously every 15 min. Furthermore, participants self-reported their diet, activity, and sleep with the help of an app ([Perfood, Germany]). Within the two weeks, two study visits took place with a duration of 7–8 h each, in which participants wore two additional sensor devices, (1) a 4-channel BiosignalsPlux explorer kit ([PLUX Biosignals, Lisbon]), that collected four sensor modalities (with a sampling rate of 200 Hz): Body temperature ‘BTEMP’, respiratory rate ‘PZT’, electrooculography ‘EOG’ and electrogastrography ‘EGG’, and (2) an Empatica E4 wristband ([Empatica, Boston), that recorded another 4 sensor modalities including skin temperature ‘STEMP’ (at 4 Hz), electrodermal activity ‘EDA’ (at 4 Hz), photoplethysmography (PPG) (producing blood volume pulse ‘BVP’ (64 Hz) and heart rate ‘HR’ (1 Hz)) (sensor placements are exhibited in Fig. 1b). Two standardized, isocaloric, isovolumetric test meals were consumed per visit in a cross-over design: a mixed meal test (MMT) (Fresubin® 2 kcal) and an oral glucose tolerance test (OGTT) (as shown in Fig. 6c and d). Participants who received the OGTT as the first test meal on their first visit received the MMT after a two-hour intervention phase and at least a two-hour washout phase, followed by another two-hour intervention phase. On their second visit, these participants received the MMT first, analogously. The choice of the first test meal (MMT or OGTT) was balanced across subjects. Before and after the different test meals, several blood, urine, and stool samples were taken for analysis of metabolome and other clinical parameters. In total, more than 1550 IG measurements and corresponding 15-min non-overlapping epochs of wearable sensor data containing the eight sensor modalities were collected. In addition, user inputs (i.e., demographic data, including age, biological sex, and BMI), time-domain inputs (seasonal information and corresponding timestamp on consumption), and food diary (standardized meal intervention) were assessed.

A follow-up study (2) was designed to validate the results of the main study under everyday conditions. Five randomly selected participants (P2b, P3b, P6b, P7b, and P10b) of the main study population participated in this follow-up study. Again, the Abbott Freestyle Libre sensor now with a higher sampling rate (every 5 min) continuously recorded their IG levels over two weeks. Throughout the two-week study period, the participants wore the Empatica E4 wristband and ate according to their individual habitual diets.

In total, over 14,400 IG measurements and corresponding 5-min non-overlapping epochs of wearable sensor data limited now only to five sensor modalities, user inputs and time-domain inputs were measured.

### Data preparation

High-quality data preprocessing is essential for robust glucose prediction, as it addresses key challenges like sensor noise, temporal misalignment, and physiological variability. Proper techniques such as artifact removal, adaptive resampling, and multimodal signal synchronization significantly improve model inputs while preserving critical glycemic patterns^60–62^.

**Fig. 7:**
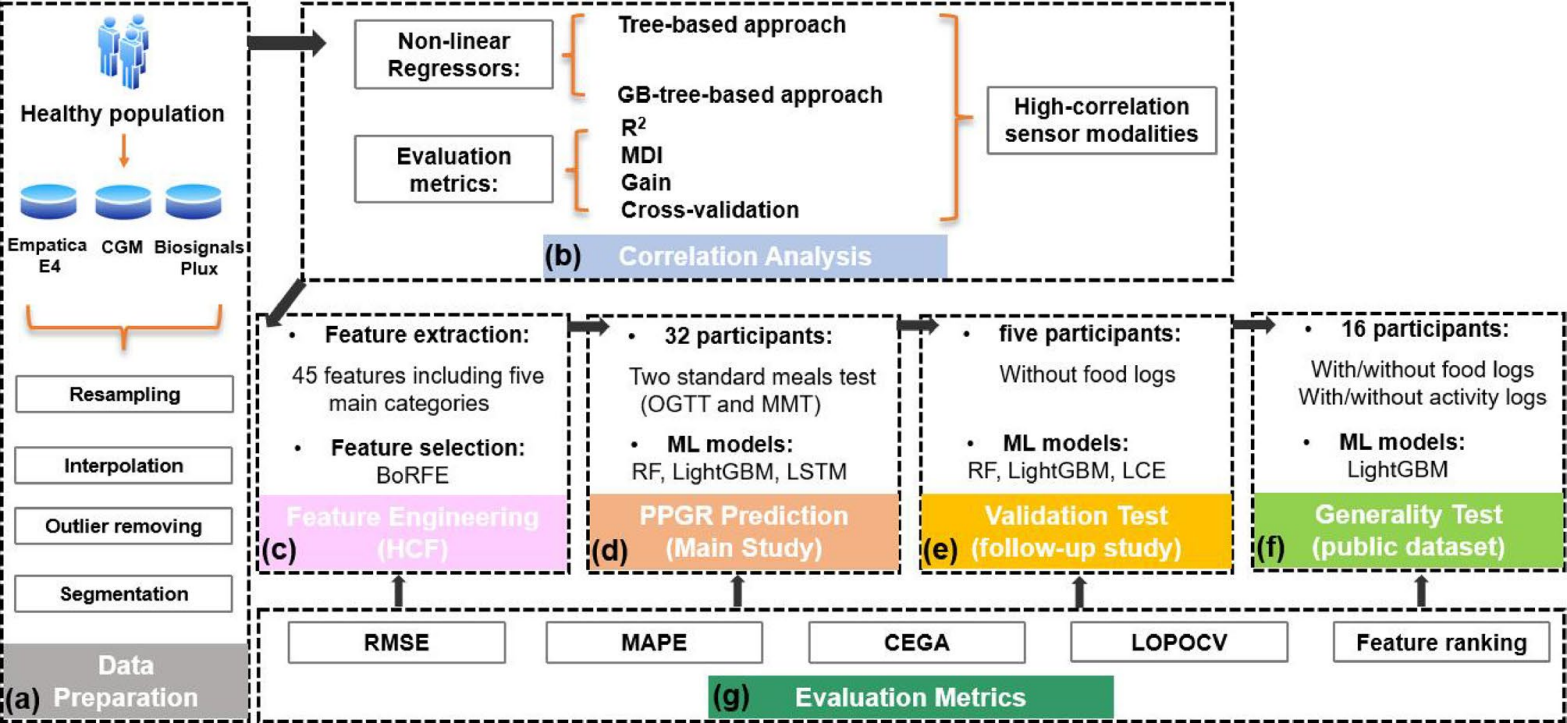
Experimental pipeline for the PPGR prediction. (**a**) Data preparation phase: four data preprocessing steps (resampling, interpolation, outliers removing, segmentation) are applied for the data collected by three types of sensor devices (Empatica E4, BiosignalsPlux, and CGM). (**b**) Correlation analysis phase: tree-based and Gradient-boost-tree-based approach are used for the non-linear correlation ablation test based on the evaluation metrics such as R^2^, MDI, Gain and cross-validation. (**c**) Feature engineering phase: 45 types of handcrafted features are extracted and then deploy the Recursive Feature Elimination and Boruta feature selection strategy to select the efficient features. (**d**) PPGR prediction based on RF, LightGBM, and LSTM models with 32 participants in the main study. (**e**) Validation test on the follow-up study dataset based on RF, LightGBM and LCE approaches with five participants without food logs. (**f**) Generality test on the BiGW public dataset with the proposed LightGBM model, which achieved the best performance from previous tests. (**g**) Evaluation metrics used for all experimental steps (**c**–**f**), including root mean square error (RMSE), mean absolute percentage error (MAPE), Clarke error grid analysis (CEGA), leave-one-participant-out cross-validation (LOPOCV), and feature ranking (FR) standard.

For this study (see Fig. 7), the following preprocessing steps were performed: resampling, denoising, and segmentation. To address temporal inconsistencies in the sensor data, including missing values, irregular sampling intervals, and varying recording frequencies across different devices, we developed a systematic data alignment protocol. The approach involved creating a master timeline with fixed, equidistant intervals determined by both the required feature sampling rate and the prediction horizon. This uniform temporal framework served two critical purposes: first, it provided a standardized reference for synchronizing all sensor data streams; second, it enabled precise temporal mapping of each data point relative to the target glucose measurements. The sensor modality data were then interpolated with a common sampling rate and their respective time information to match the predefined time steps. This method effectively normalized the temporal representation of our heterogeneous dataset while preserving the integrity of inter-signal dynamics essential for accurate glucose prediction.

After reviewing the functional subbands of all modalities, it was determined that all sensor data could be resampled to 20 Hz. Resampling reduces training runtime while maintaining performance for practical glucose prediction. Some sensors, like heart rate (1 Hz) or IG (low sampling rate), require upsampling.

Three types of interpolation approaches, such as Linear Interpolation (LiI), Nearest Neighbors’ Interpolation (NNI), and Lagrange Interpolation (LaI), were tested^63^. Considering the long PH interpolated, the advantage of LiI in the frequency domain^64^, and the requirement of computational resources, we chose to deploy the LiI method for the resampling task, except for IG. For instance, LiI was employed for sensor modalities recording such as EDA and temperature, as these parameters fall within a numerical scale range. Referring to Eq. (1), LiI is a straightforward technique used to calculate values at points that lie between known data points. Mathematical derivation involves finding a linear polynomial that intersects the unique points $\left({x}_{0},{y}_{0}\right)\text{ and }({x}_{1},{y}_{1}).$

1 $$\varepsilon_{LI} \left( x \right) = y_{0} \left( {\frac{{x - x_{1} }}{{x_{0} - x_{1} }}} \right) + y_{1} \left( {\frac{{x - x_{0} }}{{x_{1} - x_{0} }}} \right)$$

where $\varepsilon_{LI} \left( x \right)$ is commonly known as the first-order polynomial interpolation function. Due to its relatively low computational complexity, LiI is frequently used for estimating intermediate data.

In contrast, the nearest neighbor interpolation (NNI) method was tested (refers to Eq. 2) to estimate the value of a new point based on the value of the closest existing point in the dataset. Unlike more complex methods, it does not involve calculating intermediate values or derivatives. Instead, it simply finds the nearest data point and assigns its value to the new point.

2 $$\varepsilon_{NN} \left( x \right) = y_{k} \;where\;k = argmin_{i} \left| {x - x_{i} } \right|$$

Here, $\left( {xi,yi} \right)$ is a set of discrete datapoint with $i = \, 0, \, 1, \, 2, \ldots ,n$. $\varepsilon_{NN} \left( x \right)$ represents the interpolated value at any point $x$. $argmin_{i} \left| {x - x_{i} } \right|$ denotes the index k for which the distance $|x-{x}_{i}|$ is the smallest.

Notably, LiI lacks smoothness at subinterval boundaries due to its non-differentiability at these points. NNI, while simple and fast, creates a piecewise constant function that can result in significant errors between data points with large value differences. To avoid these shortcomings, for glucose-labeled data interpolated using PH as the distance between two consecutive data points, we tested Lagrange Interpolation (LaI) (see Eqs. 3 and 4), in addition to the LiI and NNI methods outlined above. Specifically, LaI generalizes linear interpolation by using higher-order polynomials (the polynomial is set to order five). For a set of n + 1 data points, the Lagrange polynomial is formulated as:

3 $$\varepsilon_{La} \left( x \right) = \sum\limits_{i = 0}^{n} {y_{i} \beta_{i} \left( x \right)}$$

where $\beta_{i} \left( x \right)$ is the Lagrange basis polynomial defined as:

4 $$\beta_{i} \left( x \right) = \prod 0 \le j \le n\frac{{x - x_{j} }}{{x_{i} - x_{j} }}, \;i \ne j$$

In addition, body temperature (BTEMP) was calculated accompanied by skin (STEMP) and core temperatures (CTEMP) refers to a proposed and verified criteria (Eq. 5) by R. Lenhardt et al.^38^ with a predefined $\alpha$ = 0.67 since our follow-up study was performed in the summer time (daylight saving time in Europe):

5 $$BTEMP = \alpha \cdot CTEMP + \left( {1 - \alpha } \right) \cdot STEMP$$

Wearable measurements may also encounter outliers due to various factors. Outliers exceeding four standard deviations from the mean are identified using Eq. (6).

6 $$x > \mu + \left( {\sigma \cdot 4} \right) or x < \mu - \left( {\sigma \cdot 4} \right)$$

where $x$ is a single sensor measurement, $\mu$ and $\sigma$ represent the mean and standard deviation across all values in a sensor modality for each participant, respectively.

Abnormal outliers in the dataset were replaced with means, using a threshold of four deviations from the mean. This choice was based on the empirical rule that 99.7% of data points lie within three standard deviations, with an additional standard deviation buffer to accommodate possible non-normal distributions in sensor data.

To prepare the data for input into ML models, the temporal information from the time series $T = \left[ {t_{0} ,t_{1} , \ldots ,t_{i - 1} } \right]$ for all sensor modalities $S = \left[ {s_{0} ,s_{1} , \ldots ,s_{n - 1} } \right]$, given by the devices and used for the experiment along with the measured data, must be defined to fit within the PH’s temporal length, represented as $p$ in min, which corresponds to the labels ${\Omega } = \left[ {\omega_{0} ,\omega_{1} , \ldots ,\omega_{m - 1} } \right]$. The sensor data from the wristband, which serves as our training data, is partitioned into equidistant time windows $J = \left[ {j_{0} ,j_{1} , \ldots ,j_{m - 1} } \right]$, with the temporal length of these windows determined by *p*. For the label $\omega_{x}$, the time interval $\left[ {t_{{\omega_{x - p} }} ,t_{{\omega_{x} }} } \right]$ forms the time window $j_{x}$ for all $S$. Depending on the length of the PH ($p$), a varying quantity of time windows arises. Consequently, when the time series length T of the label data remains constant, the quantity of time windows *J* is inversely correlative to the growth of $p.$ The formula for calculating the number of windows is shown in Eq. (7).

7 $$\left| J \right| = \left| {\Omega } \right| = \frac{{\left( {t_{{\omega_{m - 1} }} - t_{{\omega_{0} }} } \right)}}{p} + 1$$

A brief PH might lack information, while an extended one could reduce prediction performance. Therefore, a 15-min PH was chosen, with a 5-min PH for comparison purpose in experiments.

### Correlation analysis

After data preprocessing, a basic data-driven correlation ablation test was performed to comprehend the data and explore the feasibility of glucose prediction for typical meals using non-invasive sensor records. Without any FE, we adopted the tree-based (decision tree and random forest) and gradient-boosting (GB)-tree-based approaches, which are widely used and effective in various healthcare research studies^22,65–68^, with the R^2^ metric based on 32-fold cross-validation to scrutinize the inter- and intra-relationships between interstitial postprandial glucose alterations and different combinations of eight sensors. To mitigate analysis bias between specific models, decision tree and random forest were used for the tree-based category, while LightGBM and XGBoost were deployed for the GB-tree-based category to calculate the average fit score R^2^. As the appropriate research period for the glucose metabolic response is two hours post-meal, which is precisely the length of our data recording, we segmented them in terms of meal type (MMT and OGTT, excluding the washout period). Afterwards, it collaborated with polynomial-interpolation-based glucose meter values (1 value per minute) to reveal the variances and fit patterns between the given sensor modalities or their combinations and the glucose responses for a given meal type consumed in that 2-h interval. This procedure establishes a baseline for subsequent feature-driven continuous glucose prediction studies.

### Feature engineering

We developed a population predictive model based on engineered features, which can be used as a general technique to maximize the benefits of limited datasets and incorporate domain expertise into the machine learning (ML) process, with LOPOCV to predict glucose levels in healthy individuals.

A total of 45 types of features across four domains were extracted (see STable 3): demographics, statistical order, time- and frequency/amplitude-domain features, with a PH of 15 min. Z-scores^69^ were calculated (Eq. 8) to address individual variations, enabling effective prediction and calibration using both z-scores and actual measurements.

8 $${\Gamma } = \left( {x - \mu } \right)/\sigma$$

where $\Gamma$ indicates the standard z-score calculated from actual measurements $x$, mean $\mu,$ and standard deviation $\sigma.$

Forty-five features encompassed demographic data such as age, sex, and BMI, indicating biological values. These variables, with dimensions of 5 × 32 for input case (IC) in the main study and 5 × 5 for follow-up study input case 1 (FUIC1) (STable1), were kept constant per participant. Each 15-min interval contained 14 statistical order features, including mean, maximum, minimum, standard deviation, median, zero crossing, the temperature difference between STEMP and BTEMP, 20th, 50th, and 80th percentile of quartile, the interquartile difference between 25 and 75th percentile, and autocorrelations, calculated for each sensor modality (e.g., with dimensions of 14 × 5 × 32 for IC2 in the main study and 14 × 5 × 5 for FUIC1 in the follow-up study).

Furthermore, advanced signal processing metrics were applied to features such as pit and peak calculation, discrete wavelet coefficients, inverse fast Fourier transform coefficients, kurtosis, spectral energy, and spectral entropy, generating 23 frequency and amplitude domain features. These features were applied uniformly to all sensor data, resulting in dimensions of 23 × 5 × 32 for IC2 and 23 × 5 × 5 for FUIC1.

Our emphasis on time-domain features, specifically ‘minutes_from_midnight’ (current day’s minutes from midnight to encode circadian rhythm) and ‘days_from_2021’ (days from 2021’s start to calculate seasonal rhythms), addresses their significant impact on glucose levels. The former not only signifies meal timing but underscores the circadian rhythms’ physiological relevance. Additionally, ‘days_from_2021’ is crucial for accounting for seasonality. This feature accommodates variations in ambient temperatures, influenced by daylight saving time, impacting data collected by wearable sensors. These factors, alongside clustering patterns in daily life, contribute to understanding the practical implications of these features in predicting and calibrating glucose responses effectively.

As we know, diet has a large impact on glucose responses. From a physiological perspective, including meal information in glucose prediction is logical. However, in daily life, thousands of complex composition diets make quantitative and objective analysis arduous. In addition, self-reported food diaries are complex, time-consuming, and error prone. Therefore, we only implemented the food diary feature ‘consumed_food’ to facilitate the correlation between standardized meals and sensor data under predefined experimental circumstances in the main study. After the correlation was established, we eliminated the interference of food factors in our follow-up study to examine whether the sensor data could realistically reflect the underlying logic of glucose responses and whether only using sensor data could effectively predict glucose values in real-time under the unrestricted conditions of daily life.

### PPGR prediction models

In the main study, our proposed model trained and tested more than 1550 IG meter measurements and corresponding 15-min PHs from wearables in combination with a food diary (standardized meal intervention: MMT and OGTT) over a period of approximately 14 h (including washout phase) in two days across 32 participants. Subsequently, a dataset of more than 14,400 glucose meter measurements and corresponding 15-min PHs of non-invasive sensor data without food diaries and activity data measured over ten days across randomly selected five participants was exploited in the follow-up study.

We tested two regression models for predicting 15-min PHs of IG measurements based on 45 hand-crafted features implemented with various libraries (sklearn^70^ and scipy^71^) in Python with TensorFlow framework^72^ for main and follow-up studies, i.e., RF (with the configuration of hyperparameters: n_estimators = 100, max_depth = 16, n_jobs = − 1) and LightGBM (with the hyperparameters’ configuration: boosting_type = ‘gbdt’, objective = ‘regression’, num_leaves = 5000, learning_rate = 0.1, n_estimators = 100, max_depth = 16, metric = ‘mse’, bagging_fraction = 0.6, feature_fraction = 1.0, reg_lambda = 0.9, importance_type = ‘gain’). A nonFE-based recurrent neural network called LSTM was used for performance comparison. After analyzing the experimental outcomes, it was noted that the RF model performed marginally better in the main study, which involved a limited dataset. In contrast, the LightGBM predictive model was superior in processing large-scale daily data in the follow-up study with a significantly lower prediction error despite even the absence of food diaries. These experiments provided strong evidence for the effectiveness of the predesigned ensemble FS strategy that integrates the advantages of RFE and Boruta (called BoRFE), with LOPOCV and the strength of engineered features.

Typically, FS is adopted to select the effective feature set based on the contribution of each feature in the algorithm, aiming to increase the algorithm interpretability and reduce the model complexity by identifying influential features. Unfortunately, finding those optimal feature sets, especially for high-dimensional data, is a challenging task, even though many ML models are capable of generating FR. Features have a complex relationship regarding their importance to the target variable or classification, so using the features with the lowest score may not lead to optimal results. Consequently, we proposed BoRFE, which involves a two-layer FS process: Boruta effectively eliminates irrelevant features in large feature spaces. At the same time, RFE further refines the selection by iteratively removing one feature at a time collaborating with LOPOCV, which can remove the need for manual trial and error exploration. In the filtering process of BoRFE, only highly relevant hand-crafted features were always involved in the glucose prediction model’s training and optimization to ensure the experimental outcomes’ high interpretability.

### Evaluation metrics

RMSE and MAPE were used as quantitative assessment metrics for evaluating glucose prediction models for each fold of LOPOCV. Meanwhile, CEGA was utilized as a qualitative evaluation to visualize glucose prediction error distributions, which is an essential tool for examining the clinical accuracy of blood glucose self-monitoring. Its space is typically separated into five zones, i.e., A, B, C, D, and E, the brief description is as follows:

- A: Clinically correct treatment decisions.
- B: Clinically benign treatment or no treatment needs.
- C: Overcorrection.
- D: Misdiagnosing between healthy, pre-diabetes, and diabetes patients.
- E: Completely opposite or wrong diagnosis.

To enhance the interpretability of the experimental outcomes, we performed FR using two criteria: MDI and Gain (number of estimators of both models are fixed at 100), associated with the RF and LightGBM algorithms, respectively. The MDI sums and averages the actual decrease in node impurity across all trees in RF (weighted by the number of samples split), rather than counting splits. At the same time, the result of the importance calculation based on the Gain criterion includes the total gains of the splits using the feature. All features extracted from the recorded data can generally be divided into four main categories: ‘demographics’, ‘time-domain’, ‘phase marker’, and ‘sensor data’, and then further divided into 13 functional subcategories in terms of their specific functions: ‘biological age’, ‘biological sex’, ‘biological BMI (height and weight)’, ‘skin temperature’, ‘blood volume pulse’, ‘heart rate’, ‘electrodermal activity’, ‘body temperature’, ‘respiratory rate’, ‘electrogastrography’, ‘electrooculography’, ‘meal study phase’, ‘ consumption time’ and ‘seasons’. In our proposed BoRFE-based prediction model, feature importance across categories is calculated and evaluated by averaging the importance of each fold in LOPOCV. The FR results are presented as a percentage of the total significance (all importance scores are summed to 1).

## Supplementary Information

Supplementary Information.

## Data Availability

The own datasets used and/or analyzed during the current study are available from the corresponding author on reasonable request. The Big-ideas-glycemic-wearable (BiGW) dataset is available at https://physionet.org/content/big-ideas-glycemic-wearable/1.1.2/.

## References

[1] Ferrannini, E., Haffner, S. M., Mitchell, B. D. & Stern, M. P. Hyperinsulinaemia: The key feature of a cardiovascular and metabolic syndrome. *Diabetologia* **34**, 416–422 (1991). 1884900 10.1007/BF00403180

[2] Templeman, N. M., Skovsø, S., Page, M. M., Lim, G. E. & Johnson, J. D. A causal role for hyperinsulinemia in obesity. *J. Endocrinol.* **232**, 173–183 (2017). 10.1530/JOE-16-0449 28052999

[3] Prattichizzo, F. et al. Inflammageing and metaflammation: The yin and yang of type 2 diabetes. *Ageing Res. Rev.* **41**, 1–17 (2018). 29081381 10.1016/j.arr.2017.10.003

[4] Saris, W. H. M. Sugars, energy metabolism, and body weight control. *Am. J. Clin. Nutr.* **78**, 850–857 (2003). 10.1093/ajcn/78.4.850S 14522749

[5] Johnson, J. A. et al. Diabetes and cancer (1): Evaluating the temporal relationship between type 2 diabetes and cancer incidence. *Diabetologia* **55**, 1607–1618 (2012). 22476947 10.1007/s00125-012-2525-1

[6] Leon, B. M. & Maddox, T. M. Diabetes and cardiovascular disease: Epidemiology, biological mechanisms, treatment recommendations and future research. *World J. Diabetes* **6**, 1246–1258 (2015). 26468341 10.4239/wjd.v6.i13.1246 PMC4600176

[7] Ristow, M. Neurodegenerative disorders associated with diabetes mellitus. *J. Mol. Med.* **82**, 510–529 (2004). 15175861 10.1007/s00109-004-0552-1

[8] Sharma, A., Mittal, S., Aggarwal, R. & Chauhan, M. K. Diabetes and cardiovascular disease: Inter-relation of risk factors and treatment. *Futur. J. Pharm. Sci.* **6**, 1–19 (2020).

[9] Hoon Kwon, H. et al. Clinical and histological effect of a low glycaemic load diet in treatment of acne vulgaris in Korean patients: A randomized controlled trial. *Acta Derm. Venereol.* **92**, 241–246 (2012). 22678562 10.2340/00015555-1346

[10] Lelleck, V. V. et al. A digital therapeutic allowing a personalized low-glycemic nutrition for the prophylaxis of migraine: Real world data from two prospective studies. *Nutrients* **14**, 2927 (2022). 35889884 10.3390/nu14142927 PMC9315551

[11] Ley, S. H., Hamdy, O., Mohan, V. & Hu, F. B. Prevention and mangement of type 2 diabetes-dietary components and nutritional strategies. *Lancet* **383**, 1999–2007 (2014). 24910231 10.1016/S0140-6736(14)60613-9 PMC4751088

[12] Mendes-Soares, H. et al. Assessment of a personalized approach to predicting postprandial glycemic responses to food among individuals without diabetes. *JAMA Netw. Open* **2**, e188102 (2019). 30735238 10.1001/jamanetworkopen.2018.8102 PMC6484621

[13] Wang, D. D. & Hu, F. B. Precision nutrition for prevention and management of type 2 diabetes. *Lancet Diabetes Endocrinol.* **6**, 416–426 (2018). 29433995 10.1016/S2213-8587(18)30037-8

[14] Zeevi, D. et al. Personalized nutrition by prediction of glycemic responses. *Cell* **163**, 1079–1095 (2015). 26590418 10.1016/j.cell.2015.11.001

[15] Berry, S. *et al.* Personalised responses to dietary composition trial (PREDICT): An intervention study to determine inter-individual differences in postprandial response to foods. *Pre-Print* 1–23 (2020).

[16] Ben-Yacov, O. et al. Personalized postprandial glucose response—Targeting diet versus mediterranean diet for glycemic control in prediabetes. *Diabetes Care* **44**, 1980–1991 (2021). 34301736 10.2337/dc21-0162

[17] Rein, M. et al. Effects of personalized diets by prediction of glycemic responses on glycemic control and metabolic health in newly diagnosed T2DM: A randomized dietary intervention pilot trial. *BMC Med.* **20**, 56 (2022). 35135549 10.1186/s12916-022-02254-y PMC8826661

[18] Popp, C. J. et al. Effect of a personalized diet to reduce postprandial glycemic response vs a low-fat diet on weight loss in adults with abnormal glucose metabolism and obesity a randomized clinical trial. *JAMA Netw. Open* **5**, 1–13 (2023). 10.1001/jamanetworkopen.2022.33760 PMC9520362 36169954

[19] Bekkink, M. O., Koeneman, M., De Galan, B. E. & Bredie, S. J. Early detection of hypoglycemia in type 1 diabetes using heart rate variability measured by a wearable device. *Diabetes Care* **42**, 689–692 (2019). 30877089 10.2337/dc18-1843

[20] Maritsch, M. et al. Towards wearable-based hypoglycemia detection and warning in diabetes. *Ext. Abstr. 2020 CHI Conf. Hum. Factors Comput. Syst.* **152**, 1–8 (2020).

[21] Lehmann, V. et al. Noninvasive hypoglycemia detection in people with diabetes using smartwatch data. *Diabetes Care* **46**, 1–5 (2023). 10.2337/dc22-2290 PMC10154647 36805169

[22] Bent, B. et al. Engineering digital biomarkers of interstitial glucose from noninvasive smartwatches. *Npj Digit. Med.*10.1038/s41746-021-00465-w (2021). 34079049 10.1038/s41746-021-00465-w PMC8172541

[23] Lloyd, A. J. et al. Addressing the pitfalls when designing intervention studies to discover and validate biomarkers of habitual dietary intake. *Metabolomics* **15**, 1–12 (2019). 10.1007/s11306-019-1532-3 PMC6497620 31049735

[24] Ortega, R. M., Perez-Rodrigo, C. & Lopez-Sobaler, A. M. Métodos de evaluación de la ingesta actual: Registro o diario dietético. *Nutr. Hosp.* **31**, 38–45 (2015).25719769

[25] Nembrini, S., König, I. R. & Wright, M. N. The revival of the Gini importance?. *Bioinformatics* **34**, 3711–3718 (2018). 29757357 10.1093/bioinformatics/bty373 PMC6198850

[26] Adler, A. I. & Painsky, A. Feature importance in gradient boosting trees with cross-validation feature selection. *Entropy* **24**, 687 (2022). 35626570 10.3390/e24050687 PMC9140774

[27] Chiccoid, D., Onetoid, L. & Tavazzi, E. Eleven quick tips for data cleaning and feature engineering. *PLOS Comput. Biol.*10.1371/journal.pcbi.1010718 (2022). 10.1371/journal.pcbi.1010718 PMC9754225 36520712

[28] Breiman, L. Random forests. *Mach. Learn.* **45**, 5–32 (2001).

[29] Ke, G. *et al.* LightGBM: A highly efficient gradient boosting decision tree. In: *Proc. of the 31st International Conference on Neural Information Processing Systems* pp. 3149–3157 (Curran Associates Inc., 2017).

[30] Lindemann, B., Müller, T., Vietz, H., Jazdi, N. & Weyrich, M. A survey on long short-term memory networks for time series prediction. *Proc. CIRP* **99**, 650–655 (2021).

[31] Cheng, H., Garrick, D. J. & Fernando, R. L. Efficient strategies for leave-one-out cross validation for genomic best linear unbiased prediction. *J. Anim. Sci. Biotechnol.* **8**, 38 (2017). 28469846 10.1186/s40104-017-0164-6 PMC5414316

[32] Chai, T. & Draxler, R. R. Root mean square error (RMSE) or mean absolute error (MAE)?—Arguments against avoiding RMSE in the literature. *Geosci. Model Dev.* **7**, 1247–1250 (2014).

[33] Hidalgo, J. I. *et al.* Clarke and Parkes error grid analysis of diabetic glucose models obtained with evolutionary computation. In: *Proc. of the Companion Publication of the 2014 Annual Conference on Genetic and Evolutionary Computation* pp. 1305–1312 (Association for Computing Machinery, 2014). 10.1145/2598394.2609856.

[34] van den Brink, W. J., van den Broek, T. J., Palmisano, S., Wopereis, S. & de Hoogh, I. M. Digital biomarkers for personalized nutrition: Predicting meal moments and interstitial glucose with non-invasive. *Wearable Technol. Nutr.* **14**, 21 (2022). 10.3390/nu14214465 PMC9654068 36364728

[35] Jiao, Z. *et al.* A local cascade ensemble learning method for lithium ion battery SOC estimation under multi external factors considering OCV hysteresis. In: *2022 Power System and Green Energy Conference (PSGEC)* pp. 262–266 (2022). 10.1109/PSGEC54663.2022.9880963.

[36] Goldberger, A. L. et al. PhysioBank, PhysioToolkit, and PhysioNet. *Circulation* **101**, e215–e220 (2000). 10851218 10.1161/01.cir.101.23.e215

[37] Cho, P., Kim, J., Bent, B. & Dunn, J. BIG IDEAs lab glycemic variability and wearable device data. *PhysioNet* **101**, e215–e220 (2023).

[38] Lenhardt, R. & Sessler, D. I. Estimation of mean body temperature from mean skin and core temperature. *Anesthesiology* **105**, 1117 (2006). 17122574 10.1097/00000542-200612000-00011 PMC1752199

[39] Jayarathna, P., Thennakoon, K., Gunawardena, R. & Ashok, V. G. Nocturnal diabetic hypoglycemia detection using eye tracking. *In: Proc. of the 2025 Symposium on Eye Tracking Research and Applications *(2025).

[40] Barbosa, T. C., Holwerda, S. W., Young, C. N., Thyfault, J. P. & Fadel, P. J. Insulin increases respiration rate in healthy young men. *FASEB J.* **31**, lb797–lb797 (2017).

[41] Misu, N. et al. Effects of oral glucose intake on gastric myoelectrical activity and gastric emptying.. *J. Smooth Muscle Res Nihon Heikatsukin Gakkai Kikanshi* **40**(4–5), 169–176 (2004). 15655304 10.1540/jsmr.40.169

[42] Sanchez-Alavez, M. et al. Insulin causes hyperthermia by direct inhibition of warm-sensitive neurons. *Diabetes* **59**, 43–50 (2009). 19846801 10.2337/db09-1128 PMC2797943

[43] Oberle, J., Elam, M., Karlsson, T. & Wallin, B. G. Temperature-dependent interaction between vasoconstrictor and vasodilator mechanisms in human skin. *Acta Physiol. Scand.* **132**, 459–469 (1988). 3227886 10.1111/j.1748-1716.1988.tb08353.x

[44] Rosen, S. G. et al. Direct alpha-adrenergic stimulation of hepatic glucose production in human subjects. *Am. J. Physiol.* **245**(6), E616–E626 (1983). 6140854 10.1152/ajpendo.1983.245.6.E616

[45] Dolu, N., Özesmi, Ç., Çomu, N. B., Süer, C. & Gölgeli, A. Effect of hyperglycemia on electrodermal activity in diabetic rats. *Int. J. Neurosci.* **116**, 715–729 (2006). 16753897 10.1080/00207450600675027

[46] Prakash, K., Ranjan, N. & Malhotra, A. S. Blood pressure variability is better associated with acute relative hyperglycemia than the heart rate variability in healthy young adults. *Exp. Clin. Endocrinol. Diabetes* **132**, 444–451 (2023). 10.1055/a-2298-9005 38569511

[47] Yuen, K. C. J., Chong, L. E. & Riddle, M. C. Influence of glucocorticoids and growth hormone on insulin sensitivity in humans. *Diabet. Med.* **30**, 651–663 (2013). 23510125 10.1111/dme.12184

[48] Vashist, S. K. Continuous glucose monitoring systems: A review. *Diagnostics* **3**, 385–412 (2013). 26824930 10.3390/diagnostics3040385 PMC4665529

[49] Heinemann, L. et al. Benefits and limitations of MARD as a performance parameter for continuous glucose monitoring in the interstitial space. *J. Diabetes Sci. Technol.* **14**, 135–150 (2019). 31216870 10.1177/1932296819855670 PMC7189145

[50] Geng, Z., Tang, F., Ding, Y., Li, S. & Wang, X. Noninvasive continuous glucose monitoring using a multisensor-based glucometer and time series analysis. *Sci. Rep.* **7**, 12650 (2017). 28978974 10.1038/s41598-017-13018-7 PMC5627266

[51] Huang, X. et al. Comparison of feature learning methods for non-invasive interstitial glucose prediction using wearable sensors in healthy cohorts: A pilot study. *Intell. Med.* **4**, 226–238 (2024).

[52] Reddy, Y. N. R. *et al.* Machine learning approach for non-invasive detection of blood glucose concentration using microwave. In: *2018 International Conference on Advances in Computing and Communication Engineering ICACCE* pp. 89–91 (2018).

[53] Agrawal, H., Jain, P. & Joshi, A. M. Machine learning models for non-invasive glucose measurement: Towards diabetes management in smart healthcare. *Health Technol.* **12**, 955–970 (2022). 10.1007/s12553-022-00690-7 PMC9386205 35996737

[54] Aloraynan, A., Rassel, S., Xu, C. & Ban, D. A single wavelength mid-infrared photoacoustic spectroscopy for noninvasive glucose detection using machine learning. *Biosensors* **12**, 166 (2022). 35323436 10.3390/bios12030166 PMC8946023

[55] Krishnan, S. H., Vinupritha, P. & Kathirvelu, D. Non-invasive glucose monitoring using machine learning. In: *2020 International Conference on Communication and Signal Processing ICCSP* pp.780–783 (2020).

[56] Satter, S., Kwon, T.-H. & Kim, K.-D. Non-invasive blood glucose estimation based on machine learning algorithms using PPG signals. In: *International Conference on Artificial Intelligence in Information and Communication ICAIIC* pp. 622–625 (2024).

[57] Shokrekhodaei, M., Cistola, D. P., Roberts, R. C. & Quinones, S. Non-invasive glucose monitoring using optical sensor and machine learning techniques for diabetes applications. *IEEE Access* **9**, 73029–73045 (2021). 34336539 10.1109/access.2021.3079182 PMC8321391

[58] Jarvis, P. R. E., Cardin, J. L., Nisevich-Bede, P. M. & McCarter, J. P. Continuous glucose monitoring in a healthy population: Understanding the post-prandial glycemic response in individuals without diabetes mellitus. *Metab. Clin. Exp.* **146**, 155640 (2023). 37356796 10.1016/j.metabol.2023.155640

[59] Goldsack, J. C. et al. Verification, analytical validation, and clinical validation (V3): The foundation of determining fit-for-purpose for biometric monitoring technologies (BioMeTs). *Npj Digit. Med.* **3**, 55 (2020). 32337371 10.1038/s41746-020-0260-4 PMC7156507

[60] Vahedi, M. *et al.* Predicting glucose levels in patients with type1 diabetes based on physiological and activity data. In: *Proc. 8th ACM MobiHoc 2018 Workshop Pervasive Wirel. Healthc. Workshop* (2018).

[61] Mayo, M., Chepulis, L. & Paul, R. G. Glycemic-aware metrics and oversampling techniques for predicting blood glucose levels using machine learning. *PLoS ONE* **14**, e0225613 (2019). 31790464 10.1371/journal.pone.0225613 PMC6886807

[62] Gupta, M., Choudhary, M., Garg, D. & Rana, P. S. Harnessing the power of ensemble algorithms for diabetes prediction: A comparative analysis. In: *2024 IEEE International Conference on Advanced Networks and Telecommunications Systems ANTS* pp. 1–6 (2024).

[63] Jang, D.-G., Hahn, M., Jang, J.-K., Farooq, U. & Park, S.-H. A comparison of interpolation techniques for RR interval fitting in AR spectrum estimation. In: *2012 IEEE Biomedical Circuits and Systems Conference (BioCAS)* pp. 352–355 (2012). 10.1109/BioCAS.2012.6418424.

[64] Benchekroun, M. et al. The impact of missing data on heart rate variability features: A comparative study of interpolation methods for ambulatory health monitoring. *IRBM* **44**, 100776 (2023).

[65] Alfian, G. et al. Blood glucose prediction model for type 1 diabetes based on extreme gradient boosting. In *IOP Conference Series: Materials Science and Engineering* Vol. 803, 012012 (2020).

[66] Hu, L. & Li, L. Using tree-based machine learning for health studies: Literature review and case series. *Int. J. Environ. Res. Publ. Health* **19**, 16080 (2022). 10.3390/ijerph192316080 PMC9736500 36498153

[67] Ramón, A. et al. eXtreme gradient boosting-based method to classify patients with Covid-19. *J. Investig. Med.* **70**, 1472–1480 (2022). 10.1136/jim-2021-002278 35850970

[68] Hagiwara, Y. et al. Gradient boosted tree approaches for mapping European organization for research and treatment of cancer quality of life questionnaire core 30 onto 5-level version of EQ-5D index for patients with cancer. *Value Health* **26**, 269–279 (2023). 36096966 10.1016/j.jval.2022.07.020

[69] Andrade, C. Z scores, standard scores, and composite test scores explained. *Indian J. Psychol. Med.* **43**, 555–557 (2021). 35210687 10.1177/02537176211046525 PMC8826187

[70] Bisong, E. Introduction to Scikit-learn. In *Building Machine Learning and Deep Learning Models on Google Cloud Platform: A Comprehensive Guide for Beginners* (ed. Bisong, E.) 215–229 (Apress, Berkeley, 2019). 10.1007/978-1-4842-4470-8_18.

[71] Virtanen, P. et al. SciPy 1.0: Fundamental algorithms for scientific computing in python. *Nat. Methods* **17**, 261–272 (2020). 32015543 10.1038/s41592-019-0686-2 PMC7056644

[72] Abadi, M. *et al.* TensorFlow: A system for large-scale machine learning. (2016).

