# Supplementary material for: Digital biomarkers for interstitial glucose prediction in healthy individuals using wearables and machine learning
Source: Sci Rep. 2025 Aug 18;15:30164. doi: 10.1038/s41598-025-14172-z (PMC12361560; doi:10.1038/s41598-025-14172-z)
Supplement: Supplementary file 1 — Supplementary Information. [file 41598_2025_14172_MOESM1_ESM.pdf]

Digital Biomarkers for Interstitial Glucose Prediction in Healthy Individuals Using Wearables and Machine Learning

Xinyu Huang[1,†,*], Franziska Schmelter [2,†], Christian Seitzer[1], Lars Martensen[2,3], Hans Otzen[2], Artur Piet[1], Oliver Witt[3], Torsten Schröder[2,3], Ulrich L. Günther[4], Lisa Marshall[5,6], Marcin Grzegorzek[1,7,‡], Christian Sina[2,8,‡]

1. Institute of Medical Informatics, University of Luebeck, Lübeck, Germany
2. Institute of Nutritional Medicine, University of Luebeck and University Medical Center Schleswig-Holstein, Lübeck, Germany
3. Perfood GmbH, Research & Development, Lübeck, Germany
4. Institute of Chemistry and Metabolomics, University of Luebeck, Lübeck, Germany
5. Institute of Experimental and Clinical Pharmacology and Toxicology, University of Luebeck, and University Medical Center Schleswig-Holstein, Lübeck, Germany
6. Center of Brain, Behavior and Metabolism (CBBM), Lübeck, Germany
7. German Research Center for Artificial Intelligence (DFKI), Lübeck, Germany
8. Fraunhofer Research Institution for Individualized and Cell-Based Medical Engineering (IMTE), Lübeck, Germany

† These authors contributed equally to this work and shared first authorship

‡ These authors contributed equally to this work and shared last authorship

* Correspondence: hxy101726462@gmail.com

sTable 1: Definition of the different combinations of input cases in the main and follow-up studies 2
sTable 2: Availability of sensor data and demographical information of the study cohort 3
sTable 3: 45 features engineered for interstitial glucose prediction in the main and follow-up study 4

sFigure 1: Glucose prediction performance evaluation based on Clarke Error Grid Analysis (CEGA) with PH = 15 mins for four participants (naïve case - FUIC2) using BoRFE-based LightGBM regression. 7

sTable 1: Definition of the different combinations of input cases in the main and follow-up studies

Input Cases	Description
IC1	Eight sensor modalities: STEMP, BVP, EDA, HR, BTEMP, PZT, EGG, EOG
IC2	Five sensor modalities: STEMP, BVP, EDA, HR, BTEMP
IC3	Four sensor modalities from Empatica E4: STEMP, BVP, EDA, HR
IC4	Four sensor modalities from BiosignalsPlux: BTEMP, PZT, EGG, EOG
IC5	Three sensor modalities from Empatica E4: STEMP, BVP, EDA
IC6	Single sensor modality: STEMP
IC7	Single sensor modality: BVP
IC8	Single sensor modality: EDA
IC9	Single sensor modality: HR
IC10	Single sensor modality: BTEMP
IC11	Single sensor modality: PZT
IC12	Single sensor modality: EGG
IC13	Single sensor modality: EOG

FUIC1	All five participants' data (P2b, P3b, P6b, P7b, P10b) used in the follow-up study: EDA, STEMP, BTEMP, HR, and BVP
FUIC2<br><br>(naïve case)	Excluding P2b, to eliminate the risk of undiagnosed prediabetes symptoms, such as spontaneous long-term hypoglycemia: EDA, STEMP, BTEMP, HR, and BVP
FUIC3	All five participants' data by removing outliers (< 70 mg/dL): EDA, STEMP, BTEMP, HR, and BVP

sTable 2: Availability of sensor data and demographical information of the study cohort

**Participant No.**	**Sensor data (all 8 modalities) + CGM measurement**	
	**Day1**	**Day2**
**P1**	*	*
**P2**	*	*
**P3**	*	*
**P4**	*	
**P5**	*	
**P6**	*	*
**P7**	*	*
**P8**	*	*
**P9**	*	*
**P10**	*	*
**P11**	*	*
**P12**	*	*
**P13**	*	*
**P14**	*	*
**P16**		*
**P18**	*	*
**P19**		*
**P20**	*	*

**P21**	*	*
**P23**	*	
**P24**	*	*
**P27**	*	*
**P28**	*	
**P30**	*	*
**P33**	*	*
**P34**	*	*
**P35**	*	
**P36**	*	*
**P37**	*	*
**P38**	*	
**P39**	*	*
**P40**	*	
**Demographics**		
	**Women**	**Men**
**Gender (# of participants)**	22	10
	**Mean**	**SD**
**Age (years)**	26.5	4.33
**BMI (kg/m^2)**	22.7	2.63

sTable 3: 45 features engineered for interstitial glucose prediction in the main and follow-up study

**Feature source**	**Category**	**Description**
ifft_coefficientX_real	FA	Xth real coefficient of an inverse fast Fourier transformation
ifft_coefficientX_imaginary	FA	Xth imaginary coefficient of an inverse fast Fourier transformation
wavelet_dbY_approximationX	FA	Xth approximation coefficient of the discrete wavelet transform for wavelet dbX

wavelet_dbY_detailX	FA	Xth detail coefficient of the discrete wavelet transform for wavelet dbX
number_of_peaks	FA	Number of peaks
average_peak	FA	Mean average of peak sizes
median_peak	FA	Median of peak sizes
std_peak	FA	Standard deviation of peak sizes
number_of_pits	FA	Number of pits
average_pit	FA	Mean average of pit sizes
median_pit	FA	Median of pit sizes
std_pit	FA	Standard deviation of pit sizes
average_number_of_values_between_peaks	FA	Average number of values between peaks
median_number_of_values_between_peaks	FA	Median number of values between peaks
std_number_of_values_between_peaks	FA	Standard deviation for number of values between peaks
average_number_of_values_between_pits	FA	Average number of values between pits
median_number_of_values_between_pits	FA	Median number of values between pits
std_number_of_values_between_pits	FA	Standard deviation for number of values between pits
argrelextrema_number_of_peaks	FA	Number of peaks using the argrelextrema package
argrelextrema_number_of_pits	FA	Number of pits using the argrelextrema package
spectralEnergy	FA	Total amount of energy in a signal across all frequencies
spectralEntropy	FA	Metric that describes complexity of a system
kurtosis	FA	Measure of the curvature (peakiness) of the data
bio_gender	DI	Gender with 0 meaning male and 1 meaning female
bio_age	DI	Age in years
bio_height	DI	Height in cm
bio_weight	DI	Weight in kg
bio_bmi	DI	Body mass index, based on height and weight
minutes_from_midnight	TD	Number of minutes from midnight of the

		current day
days_from_2021	TD	Numbers of days from the first day of 2021
mx	SO	Maximum
mn	SO	Minimum
mean	SO	Mean average
std	SO	Standard deviation
median	SO	Median
zc	SO	Zero crossing: how frequent do the values in the time series cross 0
temperature_difference	SO	Difference between the mean average of the skin temperature and the mean average of the body temperature
pt20	SO	20th percentile: value under which 20% of values are lower
pt50	SO	50th percentile: value under which 50% of values are lower
pt80	SO	80th percentile: value under which 80% of values are lower
interquartile	SO	Difference between the 75th percentile and the 25th percentile
arg_max	SO	Index of the maximum value
arg_min	SO	Index of the minimum value
z-score	SO	Number of standard deviations of each data value from the mean
acr	SO	Auto-correlation, similarity between the time series and a delayed version of itself
consumed food (phase marker)	PM	Mark the standardized meal intervention phases: 0 is MMT, 1 is OGTT, and 2 is washout phase

Abbreviations:

FA: Frequency- and amplitude-domain feature
SO: Statistical order feature
TD: Time-domain feature
DI: Demographical information
PM: Phase marker (Standardized meal intervention phase)

sFigure 1: Glucose prediction performance evaluation based on Clarke Error Grid Analysis (CEGA) with PH = 15 mins for four participants (naive case - FUIC2) using BoRFE-based LightGBM regression.

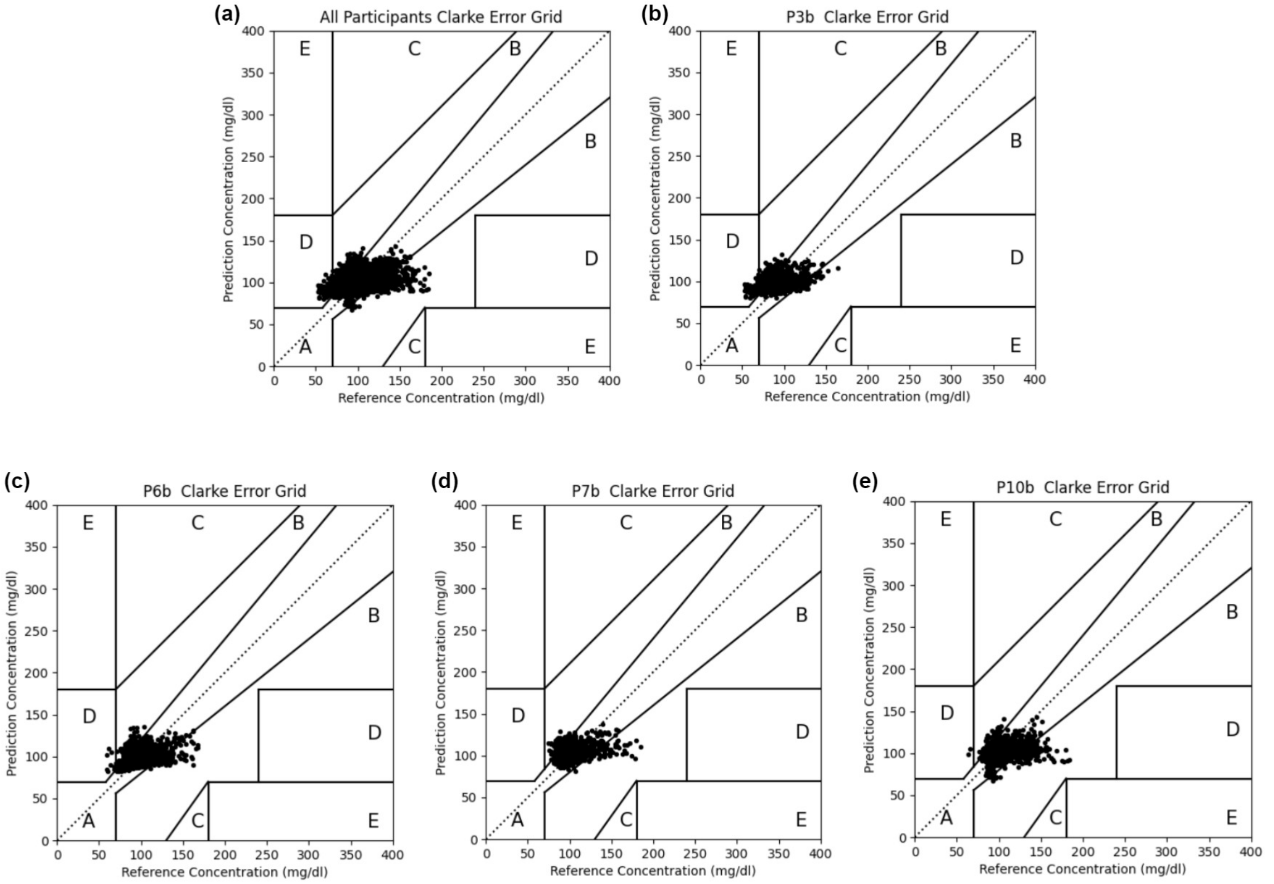

(a) Glucose prediction performance based on data from four participants assessed by CEGA with PH = 15 minutes using BoRFE-based LightGBM regression model. **(b)** Glucose prediction performance based on data from participant #3 (P3b) assessed by CEGA with PH = 15 minutes using BoRFE-based LightGBM. **(c)** Glucose prediction performance based on data from participant #6 (P6b) assessed by CEGA with PH = 15 minutes using BoRFE-based LightGBM. **(d)** Glucose prediction performance based on data from participant #7 (P7b) assessed by CEGA with PH = 15 minutes using BoRFE-based LightGBM. **(e)** Glucose prediction performance based on data from participant #10 (P10b) considered by CEGA with PH = 15 minutes using BoRFE-based LightGBM.
